# Immunization of Broiler Chickens With a Killed Chitosan Nanoparticle *Salmonella* Vaccine Decreases *Salmonella* Enterica Serovar Enteritidis Load

**DOI:** 10.3389/fphys.2022.920777

**Published:** 2022-07-18

**Authors:** Keila Acevedo-Villanueva, Gabriel Akerele, Walid Al-Hakeem, Daniel Adams, Renukaradhy Gourapura, Ramesh Selvaraj

**Affiliations:** ^1^ Department of Poultry Science, College of Agricultural and Environmental Sciences, University of Georgia, Athens, GA, United States; ^2^ Ohio Agricultural Research and Development Center, College of Food, Agricultural, and Environmental Sciences, The Ohio State University, Columbus, OH, United States

**Keywords:** Salmonella, Enteritidis, vaccines, broilers, nanoparticles

## Abstract

There is a critical need for an oral-killed *Salmonella* vaccine for broilers. Chitosan nanoparticle (CNP) vaccines can be used to deliver *Salmonella* antigens orally. We investigated the efficacy of a killed *Salmonella* CNP vaccine on broilers. CNP vaccine was synthesized using *Salmonella* enterica serovar Enteritidis (*S*. Enteritidis) outer membrane and flagella proteins. CNP was stable at acidic conditions by releasing 14% of proteins at pH 5.5. At 17 h post-incubation, the cumulative protein release for CNP was 75% at pH 7.4. Two hundred microliters of PBS with chicken red blood cells incubated with 20 μg/ml CNP released 0% hemoglobin. Three hundred chicks were allocated into 1) Control, 2) Challenge, 3) Vaccine + Challenge. At d1 of age, chicks were spray-vaccinated with PBS or 40 mg CNP. At d7 of age, chicks were orally-vaccinated with PBS or 20 μg CNP/bird. At d14 of age, birds were orally-challenged with PBS or 1 × 10^7^ CFU/bird of *S*. Enteritidis. The CNP-vaccinated birds had higher antigen-specific IgY/IgA and lymphocyte-proliferation against flagellin (*p* < 0.05). At 14 days post-infection, CNP-vaccinated birds reversed the loss in gut permeability by 13% (*p* < 0.05). At 21 days post-infection, the CNP-vaccinated birds decreased *S*. Enteritidis in the ceca and spleen by 2 Log_10_ CFU/g, and in the small intestine by 0.6 Log_10_ CFU/g (*p* < 0.05). We conclude that the CNP vaccine is a viable alternative to conventional *Salmonella* poultry vaccines.

## 1 Introduction

*Salmonella* is a zoonotic pathogen that is currently responsible for approximately four billion dollars in total costs of foodborne illness in the United States of America (United States) (Economic Research Service United States Department of Agriculture, 2021). Poultry is a core reservoir of *Salmonella* and it is linked to approximately seventy percent of salmonellosis foodborne cases (Andino and Hanning, 2015). *Salmonella* control strategies in the poultry industry consist of combined pre-harvest and post-harvest preventative strategies that aim to decrease *Salmonella* “on-farm” and also minimize the introduction of *Salmonella* at poultry processing plants when broilers reach market age. Broilers are considered to be at market age when they reach slaughter weight typically between 28 and 49 days of age (Setyohadi, Octavia and Puspitasari, 2018; Lakhmir; Singh & Manjit Kaur, 2021). One of the successful preventative strategies against *Salmonella* includes vaccinating at an early age. This strategy is facilitated by high throughput methods, like gel-spray vaccination, on the day of hatch.

For chickens, spray vaccination is a standard method for delivering respiratory vaccines, such as Newcastle Disease or Infectious Bronchitis (Tizard, 2021). Spray vaccination is especially convenient when vaccinating birds for the first time as it can be done in high throughput and an automated manner in the hatchery while the chicks are still grouped in chicken crates. Spray vaccination is one of the delivery methods that can induce mucosal immunity. For example, one way the respiratory vaccines for broilers induce mucosal immunity upon being delivered by spray is by entering the chicks’ mucosal cells in the eyes and upper respiratory tract (Nochi et al., 2018). For this study, the gel-spray method was used to deliver the CNP vaccine to newly hatched chicks. When the vaccine was sprayed on the chicks, the colored gel droplets attached to the chicks’ feathers were ingested as a form of oral vaccination when hungry newly-hatched chicks preened each other or themselves in chicken crates.

Selecting the most efficient delivery route for vaccines is vital because pathogens have different natures that allow them to thrive against the host immune defenses. For example, the oral vaccination route is key to developing mucosal immune responses against enteric pathogens, such as *Salmonella*. The oral route provides a more tailored and effective defense against *Salmonella* because systemic and mucosal immune responses are highly segregated (Li et al., 2020). However, the delivery route for conventional *Salmonella* killed vaccines for broilers are subcutaneous or intramuscular injections. The challenge is that injected vaccines induce poor mucosal immunity because they elicit specific T-cell responses in the bloodstream, resulting in predominantly IgY responses. Mucosal vaccines, however, stimulate the production of secretory immunoglobulin A (sIgA) along the gastrointestinal tract (GIT) (Lamm, 1997; Levine, 2000; Neutra and Kozlowski, 2006). The sIgA maintains homeostasis between the commensal microorganisms in the GIT and contributes to the late clearance of *Salmonella* enterica serovars from the GIT (Berthelot-Hérault et al., 2003; Forbes, Eschmann and Mantis, 2008). In addition, currently available *Salmonella* vaccines for oral delivery in broilers are live-attenuated vaccines. Live attenuated vaccines have the probability of the vaccine strain regaining its virulence (Tak W. Mak, 2006) and compromising the flock. Thus, killed *Salmonella* vaccines are preferred. Unfortunately, there are currently no commercially available oral-killed *Salmonella* vaccines for broilers. For this reason, chitosan nanoparticle (CNP) vaccines against *Salmonella* are studied as alternative vaccine candidates.

Chitosan is a natural polymer that is known for being mucoadhesive, biodegradable, non-toxic, and biologically compatible. Further, chitosan-based nanoparticles have been proved to be one of the most effective nanocarriers for the oral delivery of antigens (Imam et al., 2021). The CNP vaccine is synthesized with outer membrane proteins (OMPs) and flagella proteins extracted from *Salmonella* enterica serovar Enteritidis (Sankar Renu, Ashley D. Markazi, Santosh Dhakal, Yashavanth Shaan Lakshmanappa, Revathi Shanmugasundaram, 2018a). The synthesized CNP has (1) high cationic charge, (2) average particle size distribution of approximately 500 nm, (3) 70% encapsulation efficacy for entrapped antigens, and (4) 40% encapsulation efficacy for surface-conjugated antigens (Renu Sankar et al., 2018). In different studies, the CNP vaccine has shown to be biocompatible with broilers and layers, have no adverse effects on the production performance of broilers or layers, to successfully deliver the antigens to the Peyer’s Patches *via* oral delivery, to significantly increase the antigen-specific mucosal immune response against *Salmonella*, and to decrease *Salmonella* enterica serovar Enteritidis (*S.* Enteritidis) in the ceca (Renu, D. Markazi, et al., 2018; Acevedo-Villanueva et al., 2020; Renu, Markazi, et al., 2020a; Y. Han et al., 2020a).

Previous studies have explored the potential for mass vaccination delivery of the CNP vaccine through oral gavage, water, feed, *in-ovo*, and in a combined live followed by killed vaccination scheme (Acevedo-Villanueva et al., 2020; Renu, Han, et al., 2020; Renu, Markazi, et al., 2020; Y.; Han et al., 2020a; Yi; Han et al., 2020b; Acevedo-Villanueva et al., 2021; Acevedo-Villanueva, Renu and Gourapura, Renukaradhya, Selvaraj, 2021). Therefore, the objective of this study was to examine the efficacy of a killed *Salmonella* CNP vaccine delivered through gel-spray vaccination on broilers at d35 of age. We hypothesize that the CNP vaccine can elicit significant amounts of antigen-specific IgA and can significantly decrease the cecal and intestinal load of *S.* Enteritidis in broilers. We tested our hypothesis by (1) quantifying serum, cloacal, and bile anti-*Salmonella* OMPs IgY and IgA antibodies, (2) quantifying *S*. Enteritidis loads in the gizzard, pancreas, small intestine, spleen, liver, ceca, heart, and blood, (3) quantifying the antigen-recall response, (4) quantifying key cytokines and Toll-like receptors (TLRs) mRNA amounts, (5) quantifying fluorescein isothiocyanate dextran (FITC-d) levels in the serum, and (6) monitoring the body-weight-gain (BWG) and feed-conversion-ratio (FCR) of immunized and challenged broilers.

## 2 Materials and Methods

### 2.1 Ethical Considerations

All animal protocols were approved by the Institutional Animal Care and Use Committee (IACUC) at the University of Georgia (IACUC # A2021 04-014-Y1-A1). Experimental procedures were performed following the pertinent guidelines concerning animal handling, care, and welfare.

### 2.2 Synthesis of Chitosan Nanoparticle Vaccine

#### 2.2.1 Isolation of *S*. Enteritidis Outer Membrane Proteins

A crude protein extract of *S.* Enteritidis OMPs was used for the preparation of the CNP vaccine. The isolation of *S*. Enteritidis OMPs was done as described previously (Renu, Markazi, et al., 2020; Acevedo-Villanueva et al., 2021). In brief, a pure culture of wild-type *S.* Enteritidis was grown in Tryptic Soy Broth for 48 h at 37°C, with shaking. The grown culture was resuspended with 1 × Phosphate-Buffered Saline (PBS; pH 7.4) and centrifuged three times at 4,800 ×g for 40 min. The cell pellet was collected, washed three times using 10 mM TRIS Base buffer (pH 7.5), and heat-killed at 75 C for 20 min. The cell pellet was subsequently treated with 2% Triton X-100 in 10 mM Tris HCl buffer (pH 7.5) and disrupted using a homogenizer (OMNI Inc., GA, United States) for two rounds of 3 min each, with a cooling period on ice of 1 min in between each round. Afterward, the cell suspension was centrifuged at 4,800 ×g for 30 min and the supernatant was collected and centrifuged at 100,000 ×g for 3 h. The protein concentration was estimated using a Pierce™ BCA Protein Assay Kit (Thermo Fisher Scientific, United States), as per the manufacturer’s instructions. The final product was freeze-dried with 5% sucrose and stored until further use.

#### 2.2.2 Isolation of *S*. Enteritidis Flagellar Proteins

A crude protein extract of *S.* Enteritidis flagellin was used for the preparation of the CNP vaccine. The flagellin proteins were isolated from *S.* Enteritidis, as described previously (Renu, Markazi, et al., 2020; Acevedo-Villanueva et al., 2021). A pure culture of wild-type *S.* Enteritidis was grown in Brain Heart Infusion Broth (Sigma-Aldrich, MO, United States) for 48 h at 37°C, without shaking. The grown culture was resuspended with 1 × PBS (pH 7.4) and centrifuged three times at 4,000 ×g for 40 min. The suspension was subsequently treated with 3M Potassium Thiocyanate in 1 × PBS (pH 7.4) for 2 h at 25°C, with magnetic stirring. The treated cell suspension was centrifuged at 35,000 ×g for 30 min. The supernatant was dialyzed against 1 × PBS (pH 7.4), followed by overnight dialysis in Milli-Q water. The protein concentration was estimated using a Pierce™ BCA Protein Assay Kit, as per the manufacturer’s instructions. The end product was freeze-dried using 5% sucrose and stored until further use.

#### 2.2.3 Preparation of the Loaded Chitosan Nanoparticle Vaccine

The loaded CNP was synthesized using the ionic gelation method, as described previously (Renu, Markazi, et al., 2020; Acevedo-Villanueva et al., 2021). First, a solution of 1.0% (w/v) low molecular weight chitosan (Sigma-Aldrich, MO, United States) was made by slowly dissolving the chitosan in an aqueous solution of 4.0% acetic acid. The chitosan solution was magnetically stirred overnight, the pH was adjusted to 4.3, and the overnight stirring was repeated once more. The dissolved chitosan solution was collected and filtered using a 0.44 µm syringe filter. Afterward, 5 mL of the 1.0% chitosan solution were added to 5 ml of deionized water and magnetically stirred for 15 min. Then, the solution was incubated with 2.5 mg OMPs and flagella proteins for 15 min, with magnetic stirring. To form the nanoparticles, 2.5 ml of 1% (w/v) Sodium Tripolyphosphate (TPP) was dissolved in 2.5 ml deionized water and was subsequently added to the solution that contained the OMPs and flagella proteins, under magnetic stirring at 25°C. Finally, 2.5 mg of flagellin protein in 1 × PBS (pH 7.4) were added to the nanoparticles and the suspension was incubated for 3 h at 25 C, with magnetic stirring. The CNP vaccines were collected by centrifuging the above suspension at 10,500 ×g for 10 min. The end-product was freeze-dried using 5% sucrose and stored until further use.

#### 2.2.4 Preparation of the Loaded Chitosan Nanoparticle Vaccine in Gel-Pac Solution

The Gel-pac solution used for this study was kindly provided by Animal Science Products, Inc. and was prepared as per the manufacturer’s instructions (ASP, Inc., LOT 200507). In brief, one hundred grams of Gel-pac were dissolved into 4 L of cold water. The solution was thoroughly homogenized using a high-speed handheld emulsifier until a uniform distribution of the green coloring in the gel spray solution was obtained. For the vaccination, a total of 40 mg of loaded CNP vaccine diluted in PBS (pH 7.4) were added to the Gel-pac solution for a total volume of 500 ml stock solution. The vaccine solution was delivered in a concentration/volume of 2 mg CNP/25 ml Gel-Pac solution per box of 100 hatched chicks, to account for the delivery of 20 μg/bird. For the mock Gel-pac vaccine solution PBS (pH 7.4) was added to the Gel-pac stock solution.

#### 2.2.5 The Entrapment Efficiency of Total Proteins for Synthesized CNP Vaccine

After centrifuging the loaded CNP, the entrapment efficiency for the synthesized CNP was estimated by quantifying the amount of proteins that were left in the supernatant, as described previously (Akerele et al., 2020). In brief, the protein content in 200 μL of the supernatant was determined by using a Pierce™ BCA Protein Assay Kit, as per the manufacturer’s instructions. Afterward, the entrapment efficiency was determined as Entrapment efficiency (%) = (Total protein for the nanoparticle synthesis–Total protein left in the supernatant)/Total protein for the nanoparticle synthesis × 100.

#### 2.2.6 Cumulative Protein Release Assay and pH Stability Assay of Synthesized CNP Vaccine

The cumulative protein release of the CNP was measured by using a cumulative protein release assay, as described previously (Dhakal et al., 2018). In brief, suspensions of 0.2 mg/ml of CNP in 3 ml of 1× PBS (7.4 pH) were incubated at 37 C for 2, 3, 10, and 17 h. For each time point, a total of 350 μL of the supernatant was collected and subsequently centrifuged at 10,000 ×g at 4 C in triplicates. Two hundred microliters of the supernatant were collected, and the protein content was determined using a Pierce™ BCA Protein Assay Kit, as per the manufacturer’s instructions. The cumulative protein released for each time point was determined as Cumulative protein release (%) = (cumulative protein released in the supernatant/0.2) × 100.

A pH stability assay was used to measure the stability of the CNP at different acidic and alkaline pH, as described previously (Akerele et al., 2020). In brief, the stability of the CNP was measured by reconstituting 0.5 mg/ml of CNP in 1 × PBS at 3.5, 4.0, 4.5, 5.5, 6.5, and 7.5 pH. All the CNP suspensions were incubated at 37 °C for 6 h and subsequently centrifuged at 10,000 ×g for 5 min at 4 C. Two hundred microliters of the supernatant were collected, and the protein content was determined using a Pierce™ BCA Protein Assay Kit, as per the manufacturer’s instructions. The cumulative protein release at each pH was determined as Protein release (%) = (protein released in the supernatant/0.5) × 100.

#### 2.2.7 Hemolysis Assay- Effect of CNP Vaccine on Chicken Red Blood Cells

A hemolysis assay was done to study the biocompatibility of the CNP with chicken red blood cells (cRBCs) (Pan et al., 2016). In brief, 1 ml of blood from 4-week-old broilers was collected and centrifuged at 750 ×g to obtain the cRBCs. The cRBCs were washed four times with 1 × PBS (7.4 pH) and reconstituted in 3 ml of 1 × PBS (7.4 pH). Afterward, a total of 10 μL of cRBC suspension were incubated with 0.5 ml of 1 × PBS at 7.4 pH (negative control), pure deionized water (positive control), or 20 μg/ml, 50 μg/ml, or 100 μg/ml of CNP. All the suspensions were incubated for 3 h at 37 °C with agitation at 100 rpm. Subsequently, the suspensions were centrifuged at 750 ×g for 6 min. Two hundred microliters of the supernatant were collected, and their absorbance values were determined at 570 nm. The cRBCs hemolysis was determined as: Hemolysis (%) = (OD 595 nm Absorbance (treatment—negative control))/(OD 595 nm Absorbance (positive control−negative control)) × 100.

### 2.3 Experimental Animals

Broiler birds (Cobb-Vantress hatchery, Inc.) had access to *ad libitum* feed and water during the experimental period. Broiler birds were monitored twice a day for (1) dehydration, (2) refusal to eat food, (3) loss of body weight, (4) diarrhea, (5) bloody feces, and (6) lethargy during the experimental period. Broiler birds were euthanized with CO_2_, as per the IACUC standards.

#### 2.3.1 Treatment Groups

At d1 of age, three hundred chicks were randomly allocated into three treatment groups: 1) Control, 2) Challenge, and 3) Vaccine + Challenge. At d1 of age, all treatments were delivered using a spray cabinet (Spraycox^®^ II, K Supply Co. Inc.). Non-vaccine groups were given PBS as a mock vaccination. The vaccine group was given the CNP vaccine. At d7 of age, birds in the control and the challenge groups were given a mock booster vaccination of 0.5 ml 1 × PBS/bird, by oral gavage; while birds in the vaccine + challenge group were given a booster vaccination of 20 μg CNP/bird, by oral gavage. After vaccination, each pen was assigned 16 replicates as birds per pen. At d14 of age, birds in the control group were given a mock challenge of 0.5 ml 1 × PBS/bird by oral gavage, and birds in the challenge and the vaccine + challenge groups were orally challenged with 1 × 10^7^ CFU/bird of nalidixic acid-resistant *S*. Enteritidis. For this study, the experimental unit was the pen (*n* = 6 pen/treatment). A summary of the experimental treatment groups is provided in Table 1.

**TABLE 1:** Summary of experimental treatment groups. For all experimental groups, the experimental unit was the pen, *n* = 6 pen/treatment, with 16 technical replicates as birds/pen. For the gel-spray vaccination, 2 mg of CNP vaccine was reconstituted in 25 ml of Gel-Pac solution and sprayed on 100 chicks. For the mock gel-spray vaccination, PBS (pH 7.4) was added to the Gel-pac stock solution. For the oral gavage booster vaccination, birds in the experimental group were given 20 μg CNP/bird, and birds in the control groups were given 0.5 ml PBS/bird. At d14 of age birds in the negative control group were given a mock challenge of 0.5 ml PBS/bird and birds in the positive control and the treatment group were orally challenged with 1 × 10^7^ CFU/bird of S. Enteritidis (nalidixic acid-resistant).

Group	1^st^ Gel-spray vaccination	Oral gavage booster vaccination	Experimental challenge
Control	PBS	PBS	PBS
Challenge	PBS	PBS	1 × 10^7^ CFU/bird *S*. Enteritidis
Vaccine + Challenge	CNP	CNP	1 × 10^7^ CFU/bird *S*. Enteritidis

#### 2.3.2 Sample Collection and Preparation

In this study, the experimental unit was the pen. Treatments consisted of *n* = 6 pens per treatment, with 16 birds per pen as replicates. On the day of hatch, all chicks were screened for *Salmonella* prevalence. In brief, cloacal swab samples were enriched in Tetrathionate Broth (Neogen, MI) for 6 h. Subsequently, 10 μL of the enriched supernatant were inoculated to Modified Semi-Solid Rappaport-Vassiliadis (MSRV) Agar (Neogen, MI). Samples were subsequently incubated at 41°C for 24 h. Detection of *Salmonella* was negative on the day of hatch.

For the experimental challenge, a pure culture of wild-type *S.* Enteritidis was selected for nalidixic acid resistance on Xylose Lactose Tergitol™ 4 (XLT4) (Neogen, MI) agar at 500  mg/L. The nalidixic acid-resistant colonies were grown at 37°C for 24 h on Tryptic Soy Broth (G-Biosciences, MO, United States) containing 500 mg/L nalidixic acid and further used for the experimental challenge.

At d1 of age, one hundred birds per group were gel-spray vaccinated with either mock PBS or CNP vaccine. At 12 h post-vaccination, cecal tonsils were collected to analyze IL-1β, TNF-α, IFN-γ, IL-6, and TLR 5 mRNA expression by RT-PCR. At d7 of age, birds were treated as follows: (1) the control group and the challenge group were boosted with 1 × PBS (7.4 pH), and (2) the vaccine + challenge group was boosted with CNP vaccine. At d14 of age, birds were treated as follows: (1) the control group was given a mock challenge of 0.5 ml 1 × PBS/bird by oral gavage, and (2) the challenge group and the vaccine + challenge group were orally challenged with 1 × 10^7^ CFU/bird of nalidixic acid-resistant *S.* Enteritidis. At 12 h post-challenge, cecal tonsils were collected to analyze induced nitric oxide synthase (iNOS), IFN-γ, TNF-α, IL-10, IL-6, IL-17, TGF-β, K 60, and TLR 4 mRNA levels by RT-PCR. Bodyweight and feed consumption were recorded weekly. The BWG and FCR were calculated. Blood, bile, and cloacal swabs were collected before the experimental challenge at d14 and d35 of age. The serum, bile, and cloacal swab samples were analyzed by enzyme-linked immunosorbent assay (ELISA) for anti-OMPs IgY and IgA antibodies, respectively. At d12 of age, primary splenocytes were isolated and stimulated with either *S.* Enteritidis OMPs, *S.* Enteritidis flagellin, *S.* Enteritidis heat-killed antigen (HKA), *S.* Typhimurium HKA, *S.* Kentucky HKA, *S.* Infantis HKS, *S.* Heidelberg HKA, *S*. Hadar HKA, *S.* Litchfield HKA, or *S*. Newport HKA, to determine the recall response. At 14 days post-infection (dpi), one bird per pen was given an oral gavage of 2.2 mg FITC-d/bird, and serum was collected after 2 h for a gut permeability assay. At 21 dpi, the birds’ gizzard, pancreas, small intestine, spleen, liver, ceca, heart, and blood were collected for *S.* Enteritidis quantification by plating. At 21 dpi, cecal tonsils were collected to analyze IL-1β, TNF-α, IL-6, TGF-β, and IL-10 mRNA levels by RT-PCR, and jejunum samples were collected to analyze Claudin-1 and Zona Occludens-1 mRNA levels by RT-PCR. Spleen samples for the *ex-vivo* splenocyte recall assay were collected from two birds per pen, otherwise, samples were collected from one bird per pen (*n* = 6) at each time point.

#### 2.3.3 Antigen-Specific IgY and IgA Antibodies in Serum, Cloacal Swabs, and Bile of Vaccinated Birds

Antigen-specific IgY and IgA antibodies in serum, cloacal swab, and bile samples were assessed by ELISA, as described earlier (Renu, Markazi, et al., 2020). In brief, OMPs were diluted in 0.05 M sodium-bicarbonate coating buffer (9.6 pH) and used to coat high-binding 96-well plates (ThermoFisher Scientific, MA) with either 2 μg/ml of OMP for IgG or 7.5 μg/ml of OMP for IgA (Renu, Markazi, et al., 2020). The OMP-coated plates were incubated overnight at 4°C, with no shaking. The incubated plates were washed three times with 0.05% PBS-Tween 20 (PBS-T; pH 7.4) and were subsequently blocked with 5% non-fat dry milk powder in PBS-T for 1 h at 37^°^C. The unbound antigens were removed by washing the plates three times with PBS-T. A two-fold serial dilution for a total of 100 μL per well was carried out for each sample. Serum and bile samples were diluted with 2.5% non-fat dry milk and cloacal swabs samples were diluted in 1 × PBS (pH 7.4). Negative serum was used as a control for serum samples, negative bile was used as a control for bile samples, and sterile 1 × PBS (pH 7.4) was used as a control for cloacal swabs samples. The samples were incubated for 2 h at 37^°^C and subsequently washed three times with PBS-T. Fifty microliters of the HRP-conjugated goat anti-chicken IgG (Southern Biotech, AL) were added at 1: 10,000 in 2.5% non-fat dry milk powder in PBS-T or 50 μL of the HRP-conjugated goat anti-chicken IgA (Bethyl Laboratories, TX) were added at 1: 3,000 in 2.5% skim milk powder in PBS-T. The secondary antibodies were incubated for 2 h at 37°C. Plates were subsequently washed three times with PBS-T, and 50 μL per well of TMB peroxidase substrate (KPL, MD) were added. After 5 min, the reaction was stopped by adding 50 μL per well of 2M Sulfuric Acid (J.T. Baker Inc., NJ, United States). The Optical Density (OD) was measured at 450 nm using a spectrophotometer and the corrected OD was calculated by subtracting the treatment OD from the blank OD. Results were reported as geometric mean titers (GMT). The cut-off values were determined by the mean (x) and standard deviation (SD) of the negative sera for serum samples, negative bile for bile samples, and PBS controls for cloacal swabs samples. The cut-off value was taken as x + 3SD, as described previously (Lunn et al., 2012). The GMT was calculated with the use of the log-transformed values and taken as the antilog of the mean of the transformed values, as described previously (Perkins, 1958; Belshe et al., 2004). The percent increase was determined as [(GMT of Vaccine + Challenge)—(GMT of Control)] ÷ (GMT of Control) × 100.

#### 2.3.4 Recall-Response of Spleenocytes of Vaccinated Birds

For this study, the spleen samples were collected from two birds per pen (*n* = 6) at d12 of age. The recall-response was determined using an *ex-vivo* recall assay, as previously described (Acevedo-Villanueva et al., 2021). Briefly, the whole spleen was passed through a cell strainer with 3 ml of sterile 1 × PBS (7.4 pH) to obtain a single-cell suspension of PBMCs. The single-cell suspension was slowly added onto 3 ml of Ficoll-paque plus solution (Fisher Scientific, MA, United States). To remove the red blood cells the suspension was centrifuged at 450 × g for 30 min at 4°C. Subsequently, the splenocytes in the interface were slowly harvested. The cells were reconstituted using 100 μL of RPMI-1640 (Sigma Aldrich, MO, United States) supplemented with 10% fetal bovine serum and 1% Penicillin and Streptomycin. The cell suspension was plated at 5 × 10^6^ cells per well in duplicates. For the first recall response assay, the cells were stimulated with 20 μg/ml of OMPs crude protein extract or 20 μg/ml of flagellin crude protein extract and incubated for 3 days at 37°C in the presence of 5% CO_2_. For the second recall response assay, cells were stimulated with 20 μg/ml of either *S.* Enteritidis HKA, *S.* Typhimurium HKA, *S.* Kentucky HKA, *S.* Infantis HKS, *S.* Heidelberg HKA, *S*. Hadar HKA, *S.* Litchfield HKA, or *S*. Newport HKA. The HKA was prepared by boiling the bacterial stock for 15 min, sonicating to make soluble antigen, and centrifuging at 1,000 ×g for 15 min to obtain the bacterial suspension. The protein concentration was assessed using the Pierce™ BCA Protein Assay Kit, as per the manufacturer’s instructions. Further, a Sodium Dodecyl Sulphate–Polyacrylamide Gel Electrophoresis (SDS-PAGE) analysis was done to visualize the *S*. Enterica serovars heat-killed whole-antigenic crude extract (Supplementary Figure S1). Before stimulation, the bacterial suspension was re-suspended in the enriched RPMI-1640. As a negative control for both recall response assays, splenocytes were stimulated with 0.0 μg/ml proteins. As a positive control for both recall response assays, splenocytes were stimulated with 20 μg/ml of Concanavalin A (Con A). The proliferation of spleenocytes was measured using an MTT assay, as described previously (Zhao et al., 2012). The optical density was measured at 570 nm using a spectrophotometer.

#### 2.3.5 *Salmonella* Loads in the Gizzard, Pancreas, Small Intestine, Spleen, Liver, Ceca, Heart, and Blood of Vaccinated Birds

Gizzard, pancreas, small intestine, spleen, liver, ceca, heart, and blood samples were analyzed for *S.* Enteritidis loads by plating. All feed was removed from organs by washing with approximately 3 ml of 1 × PBS (pH 7.4). All samples were stored in stomacher bags and placed on ice. At the laboratory, samples were diluted with 1× (wt/vol) 1 × PBS (pH 7.4), mashed with a rubber mallet, and then stomached for 2 min. A volume of 100 μL of ceca was serially diluted into 900 μL of 1 × PBS (pH 7.4) and from every dilution, a volume of 10 μL was plated in duplicates on XLT4 agar plates. Plates were then incubated for 24 h at 41°C for the confirmation of black colonies. When no growth was observed, to corroborate true negative samples, the samples were further enriched in Tetrathionate Broth for 6 h followed by the inoculation of 10 μL of the enriched solution to XLT4 agar or MSRV Agar. The inoculated media were then incubated for 24 h at 41°C for the confirmation or the absence of black colonies on XLT-4 agar plates or the confirmation of positive or negative samples on MSRV selective motility-enrichment media. Upon double confirmation of the absence of growth, the samples were considered to be negative for *Salmonella* colonization. Data were recorded as CFU/g of organ and then transformed to Log 10 CFU/g of organ for statistical analysis. Further, the prevalence of *S.* Enteritidis in colonized organs was also calculated. In addition, the organ weight was further used to observe the CNP vaccine effect on the relative weight of different organs by calculating the organ index as Weight Index (%) = (organ weight (g))/(live weight (g)) × (100).

#### 2.3.6 FITC-d Concentration in the Serum of Vaccinated Birds

To determine the serum FITC-d levels at 14 dpi, one bird per pen was given FITC-d (MW 3–5 kDa; Sigma-Aldrich Co., St. Louis, MO, United States) by oral gavage at a dose of 2.2 mg FITC-d per bird (Liu et al., 2021). After 2 h the chicks were euthanized by CO_2_ inhalation and blood samples were collected. As a control, serum was taken from one broiler chicken of each treatment group that was not given FITC-d. The blood samples were collected and centrifuged at 3,000 rpm for 12 min at 4°C. The serum was then collected and diluted at 1:5. The OD was measured at 485 nm using a spectrophotometer.

#### 2.3.7 Gene Expression in the Cecal Tonsils or Jejunum of Vaccinated Birds

Cecal tonsils were collected to monitor the mRNA expression of key cytokines of the gut-associated lymphoid tissue. Jejunum samples were also collected to monitor the expression of key cytokines of tight-junction proteins in the gut. For this study, cecal tonsil and jejunum samples were collected from one bird per pen (*n* = 6) at 21 dpi. For gene expression analysis samples were analyzed in duplicates. The TRIzol reagent (Invitrogen, CA, United States) was used for the total RNA extraction, as per the manufacturer’s instructions. The extracted RNA was dissolved in Tris-EDTA buffer (pH 7.5) and the cDNA synthesis was executed using 2 µg of total RNA template in a 20 µL reaction volume, as described previously (Acevedo-Villanueva et al., 2021). The cecal tonsils mRNA transcripts were analyzed for IL-1β, TNF-α, IFN-γ, IL-6, and TLR 5 mRNA levels at 12 h post-vaccination, or analyzed for iNOS, IFN-γ, TNF-α, IL-10, IL-6, IL-17, TGF-β, K 60, and TLR 4 mRNA levels at 12 h post-challenge, or analyzed for IL-1β, TNF-α, IL-6, TGF-β, and IL-10 mRNA levels at 21 dpi by RT-PCR (CFX96 Touch Real-Time System, BioRad). The jejunum mRNA transcripts were analyzed for Claudin-1 and Zona Occludens-1 mRNA levels at 21 dpi by RT-PCR. All reactions were carried out using iQ™ SYBR® Green Supermix (ThermoFisher Scientific, MA), as described previously (Acevedo-Villanueva et al., 2021). The housekeeping gene Ribosomal Protein S13 (RPS13) was used as a reference gene to normalize the Ct values (de Jonge et al., 2007). The fold change from the reference was determined using the delta-delta Ct method, as explained previously (Schmittgen and Livak, 2008). Results were reported as the fold-change (2^−ΔΔCt^ method). The primers sequences used for RT-PCR analysis are described in Table 2.

**TABLE 2:** Primers and PCR conditions for RT-PCR.

Target Gene	Sequence (5’—3′)	T_a_ (°C)	Reference
IL-1β (F)	TCC​TCC​AGC​CAG​AAA​GTG​A	57.0	Morris et al. (2014)
IL-1β (R)	CAG​GCG​GTA​GAA​GAT​GAA​GC		
IFN-γ (F)	GTG​AAG​AAG​GTG​AAA​GTA​TCA​TGG​A	57.0	Kaiser, Underwood and Davison, (2003)
IFN-γ (R)	GCT​TTG​CGC​TGG​ATT​CTC​A		
IL-10 (F)	CATGCTGCTGGGCCTGAA	57.5	Rothwell et al. (2004)
IL-10 (R)	CGT​CTC​CTT​GAT​CTG​CTT​GAT​G		
iNOS (F)	AGT​GGT​ATG​CTC​TGC​CTG​CT	60.0	Selvaraj and Klasing, (2006)
iNOS (R)	CCA​GTC​CCA​TTC​TTC​TTC​C		
TGF-β (F)	AGG​ATC​TGC​AGT​GGA​GTG​GAT	54.0	Han et al. (2020b)
TGF-β (R)	CCCCGGGTTGTGTTGGT		
IL-6 (F)	CAA​GGT​GAC​GGA​GGA​GGA​C	57.5	Hong et al. (2012)
IL-6 (R)	TGGCGAGGAGGGATTTCT		
TNF-α (F)	ATC​CTC​ACC​CCT​ACC​CTG​TC	56.0	Han et al. (2020b)
TNF-α (R)	GGC​GGT​CAT​AGA​ACA​GCA​CT		
IL-17 (F)	GCA​GAT​GCT​GGA​TGC​CTA​AC	55.5	Markazi et al. (2018)
IL-17 (R)	ATG​GAG​CCA​GTG​AGC​GTT​T		
TLR 4 (F)	ACC​TAC​CCA​TCG​GAC​ACT​TG	60.0	Markazi, et al. (2018)
TLR 4 (R)	TGC​CTG​AGA​GAG​GTC​AGG​TT		
TLR 5 (F)	CCT​TGT​GCT​TTG​AGG​AAC​GAG​A	52.3	Xu et al. (2015)
TLR 5 (R)	CAC​CCA​TCT​TTG​AGA​AAC​TGC​C		
K60 (F)	ATT​TCC​TCC​TGC​CTC​CTA​CA	55.0	Hong et al. (2006)
K60 (R)	GTG​ACT​GGC​AAA​AAT​GAC​TCC		
Claudin-1 (F)	TGT​AGC​CAC​AGC​AAG​AGG​TG	55.0	Chen et al. (2017)
Claudin-1 (R)	GAC​AGC​CAT​CCG​CAT​CTT​CT		
Zona Occludens-1 (F)	TGT​AGC​CAC​AGC​AAG​AGG​TG	55.0	Oxford and Selvaraj, (2019)
Zona Occludens-1 (R)	CTG​GAA​TGG​CTC​CTT​GTG​GT		
RPS13 (F)	CAA​GAA​GGC​TGT​TGC​TGT​TCG	55.5	Hutsko. (2017)
RPS13 (R)	GGC​AGA​AGC​TGT​CGA​TGA​TT		

### 2.4 Statistical Analysis

The experimental unit was the pen, where *n* = 6 pens per treatment, with 16 replicates as birds per pen. At each time point, samples were taken from one bird per pen, except for the spleen samples, which were taken from two birds per pen. For this study, all the samples were analyzed in duplicates. Data for the antigen-specific recall response of immunized birds against different *Salmonella* enterica serovars HKA was analyzed by parametric Student t-test. For the multiple comparisons of other data, if data were normally distributed the analysis was done using a one-way analysis of variance (ANOVA), followed by a Tukey’s post-hoc test. Otherwise, the statistical differences were determined using a Kruskal-Wallis test and followed by Dunn’s post-hoc test. Statistical analysis was performed using JMP Pro 14 (SAS Institute Inc., United States) and results were statistically significant at *p* < 0.05.

## 3 Results

### 3.1 *In-vitro* Analysis of Synthesized CNP Vaccine

#### 3.1.1 The Entrapment Efficiency of Total Proteins for Synthesized CNP Vaccine

The entrapment efficiency for the total protein content of the CNP vaccine was 87% (data not shown).

#### 3.1.2 The pH Stability Assay and Cumulative Protein Release Assay of Synthesized CNP Vaccine

At 6 h post-incubation the CNP released 3, 9, 10, 14, 31 and 26% of proteins from 3.5 to 7.5 pH, respectively (Table 3).

**TABLE 3:** The pH stability assay and cumulative protein release assay of the synthesized CNP vaccine. The CNP was incubated in 1 × PBS at multiple pH’s for 6 h.

pH stability of the CNP vaccine	
**pH**	**Protein release (%)**
3.5	3
4.0	9
4.5	10
5.5	14
6.5	31
7.5	26

At 2 h post-incubation the CNP had released 11% of its protein cargo, at 3 h post-incubation the CNP had released 14% of its protein cargo, at 10 h post-incubation the CNP had released 21% of its protein cargo, and at 17 h post-incubation the CNP had released 75% of its protein cargo (Table 4).

**TABLE 4:** The pH stability assay and cumulative protein release assay of the synthesized CNP vaccine. The CNP were incubated in 1 × PBS at 7.4 pH and the cumulative antigen release was assessed at different time points. Means +SD. *n* = 2.

Cumulative protein release of the CNP vaccine	
**Hours**	**CPR (%)**
2	11
3	14
10	21
17	75

#### 3.1.3 Hemolysis Assay- Effect of CNP Vaccine on Chicken Red Blood Cells

The cRBC incubated with 20 μg/ml, 50 μg/ml, and 100 μg/ml of CNP had 0.07, 0.09 and 0.9% hemolysis respectively (Table 5).

**TABLE 5:** Effect of CNP on chicken red blood cells. The CNP was synthesized by entrapping a crude-enriched extract of OMP and Flagellin proteins from *Salmonella* enterica serovar Enteritidis. Mean ± SD. *n* = 2.

cRBCs hemolysis (%)		
20 μg/ml	50 μg/ml	100 μg/ml
0.07 ± 0.030	0.09 ± 0.007	0.94 ± 0.021

### 3.2 The Effects of *Salmonella* CNP Vaccine on the Production Performance of Vaccinated Birds

The CNP vaccine had no adverse effect on the production performance of the vaccinated birds. There were no significant differences (*p* > 0.05) between treatments in the BWG or FCR of birds at all the time points, compared to control; hence, results were reported as cumulative BWG (Supplementary Figure S2A) and FCR from d0 to d35 of age (Supplementary Figure S2B).

### 3.3 The Effects of *Salmonella* CNP Vaccine on Antigen-Specific IgY and IgA Antibodies of Vaccinated Birds

At d14 of age, the birds that were vaccinated with CNP had significantly increased anti-*S*. Enteritidis OMP IgY serum titers by 218%, when compared to the control group (*p* < 0.05) (Table 6). At d35 of age, there were no significant differences in anti-*S*. Enteritidis OMP IgY titers from serum samples between any of the treatment groups when compared to the control group (*p* = 0.05) (Table 6). At d14 of age, the birds that were vaccinated with CNP had significantly increased anti-*S*. Enteritidis OMP IgA bile titers by 535%, when compared to the control group (*p* < 0.05) (Table 6). At d35 of age, the birds that were vaccinated with CNP had significantly increased anti-*S*. Enteritidis OMP IgA bile titers by 535%, when compared to the control group (*p* < 0.05) (Table 6). At d14 of age, there were no significant differences in anti-*S*. Enteritidis OMP IgA titers from cloacal swab samples between any of the treatment groups when compared to the control group (*p* = 0.05) (Table 6). At d35 of age, the birds that were vaccinated with CNP had significantly increased anti-*S*. Enteritidis OMP IgA cloacal titers by 1033%, when compared to the control group (*p* < 0.05) (Table 6).

**TABLE 6:** The GMT of anti-*S.* Enteritidis OMP IgY and IgA antibodies. Blood, bile, and cloacal swabs samples were collected at d14 of age (pre-challenge) and d35 of age (post-challenge). Samples were analyzed for anti-*Salmonella* OMP IgY and OMP IgA levels by ELISA (*n* = 6). Results were reported as geometric mean titers (GMT). Values with no common superscript differ (*p* < 0.05). The percent increase was determined as [(GMT of Vaccine + Challenge)–(GMT of Control)] ÷ (GMT of Control) × 100.

OMP					
Sample	Day of age	Treatment group	GMT	% CV	*p*-value
Serum	d14	Control	228^b^	69	*p* < 0.05
		Challenge	128^ab^	79	
		Vaccine + Challenge	724^a^	49	
	d35	Control	144	20	*p* = 0.05
		Challenge	1448	42	
		Vaccine + Challenge	1290	47	
Bile	d14	Control	645^c^	82	*p* < 0.05
		Challenge	724^b^	63	
		Vaccine + Challenge	4096^a^	0	
	d35	Control	512^b^	59	*p* < 0.05
		Challenge	4096^a^	0	
		Vaccine + Challenge	3250^a^	31	
Cloacal swabs	d14	Control	23	67	*p* = 0.05
		Challenge	51	20	
		Vaccine + Challenge	2298	52	
	d35	Control	161^b^	89	*p* < 0.05
		Challenge	2580^a^	49	
		Vaccine + Challenge	1824^a^	68	

The meaning of the symbol for (a, b) in Table is in indicated in the figure legend as “Values with no common superscript differ (*p* < 0.05).” It indicates the significance of the data.

At d14 of age and d35 of age, there were no significant differences in anti-*S*. Enteritidis Flagellin IgY titers from serum samples between any of the treatment groups when compared to control (*p* > 0.05) (Table 7). At d14 of age, the birds that were vaccinated with CNP had significantly increased anti-*S*. Enteritidis Flagellin IgA bile titers by 218%, when compared to the control group (*p* < 0.05) (Table 7). At d35 of age, the birds that were vaccinated with CNP had significantly increased anti-*S*. Enteritidis Flagellin IgA bile titers by 256%, when compared to the control group (*p* < 0.05) (Table 7). At d14 of age, there were no significant differences in anti-*S*. Enteritidis Flagellin IgA titers from cloacal swabs samples between any of the treatment groups when compared to control (*p* > 0.05) (Table 7). At d35 of age, the birds that were vaccinated with CNP had significantly increased anti-*S*. Enteritidis Flagellin IgA cloacal titers by 1710%, when compared to the control group (*p* < 0.05) (Table 7).

**TABLE 7:** The GMT of anti-*S.* Enteritidis Flagellin IgY and IgA antibodies. Blood, bile, and cloacal swabs samples were collected at d14 of age (pre-challenge) and d35 of age (post-challenge). Samples were analyzed for anti-*Salmonella* Flagellin IgY and Flagellin IgA levels by ELISA (*n* = 6). Results were reported as geometric mean titers (GMT). Values with no common superscript differ (*p* < 0.05). The percent increase was determined as [(GMT of Vaccine + Challenge)–(GMT of Control)] ÷ (GMT of Control) × 100.

Flagellin					
Sample	Day of age	Treatment group	GMT	% CV	*p*-value
Serum	d14	Control	512	0	*p* > 0.05
		Challenge	181	70	
		Vaccine + Challenge	1024	56	
	d35	Control	574	35	*p* > 0.05
		Challenge	1024	59	
		Vaccine + Challenge	812	75	
Bile	d14	Control	456^b^	68	*p* < 0.05
		Challenge	813 ^ab^	72	
		Vaccine + Challenge	1448^a^	37	
	d35	Control	512^b^	0	*p* < 0.05
		Challenge	2047^a^	0	
		Vaccine + Challenge	1824^a^	22	
Cloacal swabs	d14	Control	57	34	*p* > 0.05
		Challenge	18	32	
		Vaccine + Challenge	91	30	
	d35	Control	80^b^	82	*p* < 0.05
		Challenge	813^a^	70	
		Vaccine + Challenge	1448^ab^	85	

The meaning of the symbol for (a, b) in Table is in indicated in the figure legend as “Values with no common superscript differ (*p* < 0.05).” It indicates the significance of the data.

### 3.4 The Effects of *Salmonella* CNP Vaccine on the Antigen Recall Response of Vaccinated Birds

**FIGURE 1:**
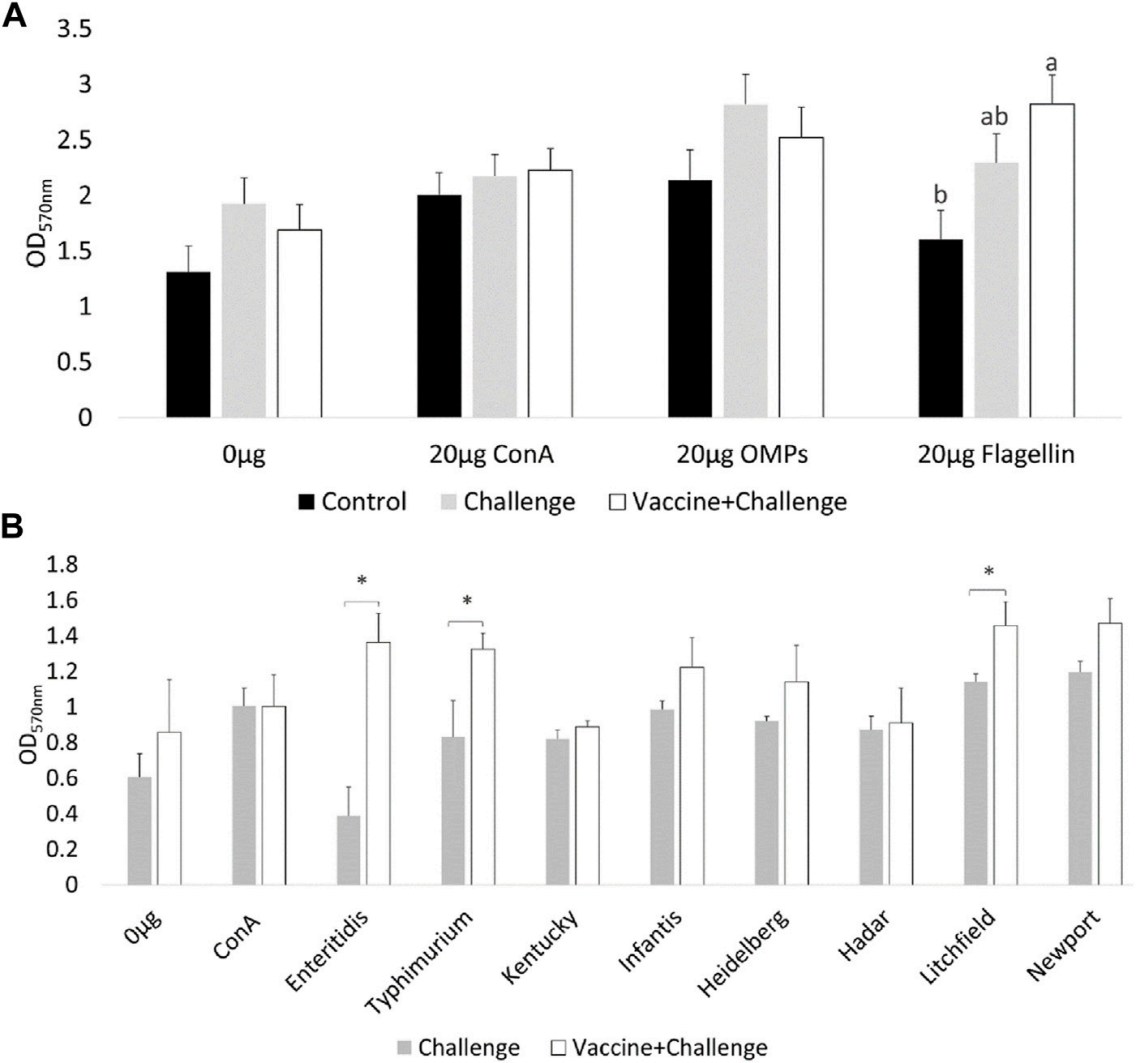
*Ex-vivo* recall-response of spleenocytes of vaccinated birds. At d12 of age, splenocytes PBMCs were stimulated with either 20 μg/ml OMP, 20 μg/ml Flagellin or 20 μg/ml proteins of different *S*. Enterica serovars HKA for 3 days. As a negative control splenocytes were stimulated with 0.0 μg/ml of antigen. As a positive control splenocytes were stimulated with 20 μg/ml Con A. **(A)** OMP and Flagellin. Bars (+SE) with no common superscript differ (*p* < 0.05); **(B)** HKA from *S.* Enterica serovars. Means + SE Bars. *n* = 6. “*” signifies *p* < 0.05.

At d12 of age, the spleenocytes from birds that were immunized with the CNP vaccine had significant (*p* < 0.05) T-lymphocyte proliferation when they were stimulated with 20 μg/ml Flagellin, compared to control (Figure 1A). There were no significant differences in T-lymphocyte proliferation when the spleenocytes were stimulated with 20 μg/ml OMP, compared to control (Figure 1A).

At d12 of age, the spleenocytes from birds that were immunized with the CNP vaccine had significant (*p* < 0.05) T-lymphocyte proliferation when they were stimulated with 20 μg/mL *S*. Enteritidis HKA, 20 μg/mL *S.* Typhimurium HKA, and 20 μg/mL *S.* Litchfield HKA, compared to control (Figure 1B).

### 3.5 The Effects of *Salmonella* CNP Vaccine on *Salmonella* Loads in Gizzard, Pancreas, Small Intestine, Spleen, Liver, Ceca, Heart, and Blood of Vaccinated Birds

**FIGURE 2:**
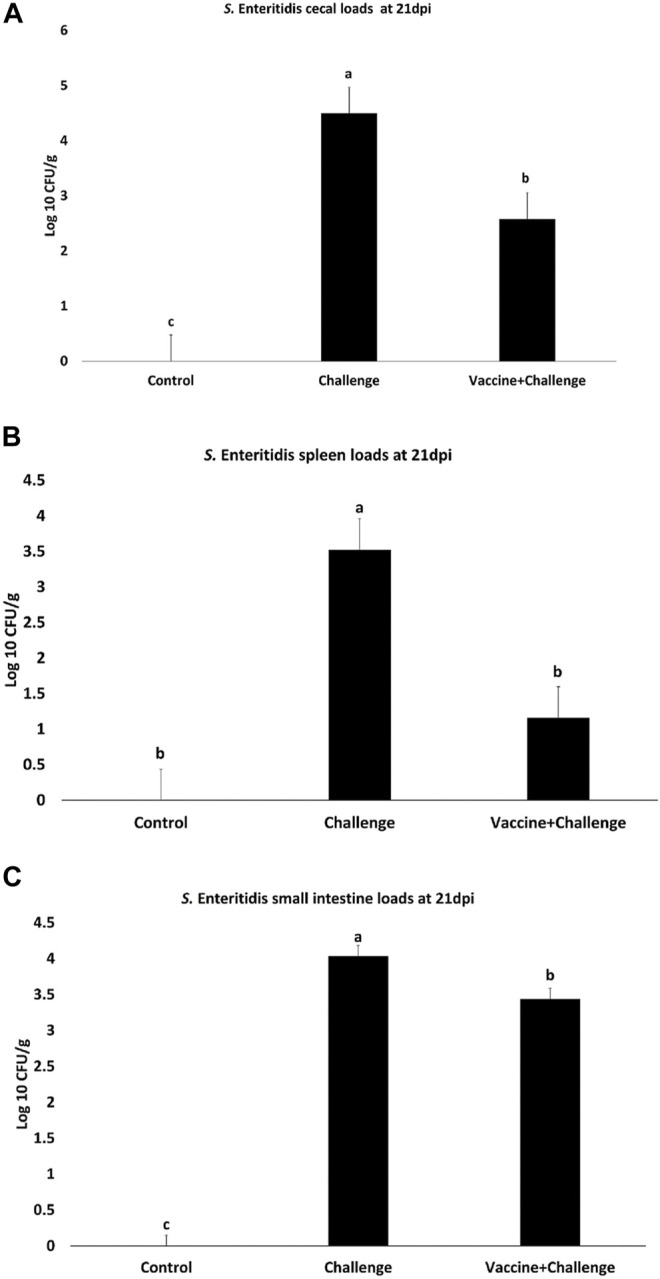
*Salmonella* loads in the ceca, spleen, and small intestines of vaccinated birds. Organ samples were collected from 1 bird per pen (*n* = 6) at 21 dpi. Samples were analyzed for *S*. Enteritidis loads by plating. Samples that were double negative for *S*. Enteritidis presence after selective enrichment were considered negative for *Salmonella* colonization. Data were recorded as CFU/g of organ and transformed to Log 10 CFU/g of organ for statistical analysis. **(A)** Ceca; **(B)** Spleen; **(C)** Small intestine. Bars (+SE) with no common superscript differ (*p* < 0.05).

At 21 dpi the CNP-vaccinated birds had a 2 Log_10_ CFU/g, 2 Log_10_ CFU/g, 0.6 Log_10_ CFU/g decrease in *S*. Enteritidis loads in the ceca (Figure 2A), spleen (Figure 2B), and small intestine (Figure 2C), respectively, compared to that in the control (*p* < 0.05). There was no *S*. Enteritidis detection in gizzard, pancreas, liver, heart, and blood when compared to control (*p* > 0.05).

The prevalence of *S*. Enteritidis in the ceca of the immunized birds was 33% for *Salmonella* negative birds and 67% for *Salmonella* positive birds (Supplementary Figure S3A). The prevalence of *S*. Enteritidis in the spleen of the immunized birds was 67% for *Salmonella* negative birds and 33% for *Salmonella* positive birds (Supplementary Figure S3B). The prevalence of *S*. Enteritidis in the small intestine of the immunized birds was 17% for *Salmonella* negative birds and 83% for *Salmonella* positive birds (Supplementary Figure S3C).

Further, there were no significant differences between any treatment groups for the organ index when compared to control (*p* > 0.05) (Supplementary Figure S4).

### 3.6 The Effects of *Salmonella* CNP Vaccine on FITC-d Concentration in the Serum of Vaccinated Birds

**FIGURE 3:**
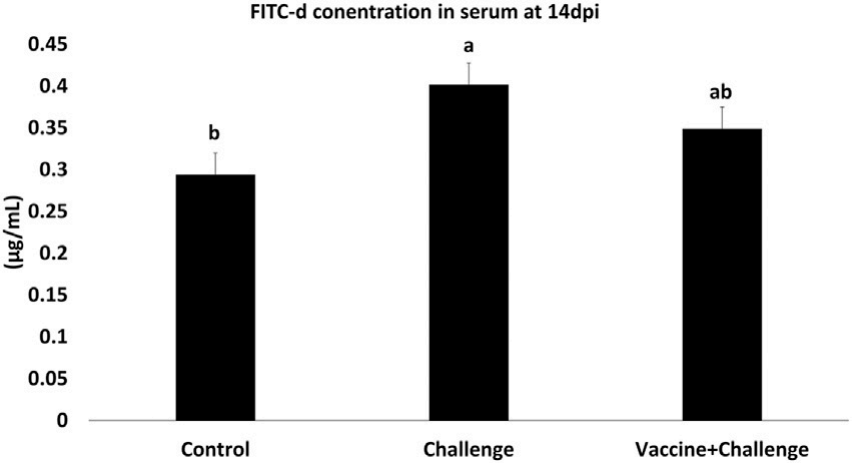
FITC-d concentration in the serum of vaccinated birds. At d1 of age birds were allocated into treatment groups: 1) Control; 2) Challenge; or 3) Vaccine + Challenge. At d1 and d7 of age birds in the negative and positive control groups were mock vaccinated with PBS and birds in the treatment group were vaccinated with CNP. At d14 of age birds in the negative control group were given a mock challenge of 0.5 ml PBS/bird and birds in the positive control and the treatment group were orally challenged with 1 × 10^7^ CFU/bird of *S*. Enteritidis. At 14 dpi, one bird per pen was given 2.2 mg FITC-d by oral gavage. After 2 h blood samples were collected. The Optical Density (OD) was measured at 485 nm. Bars (+SE) with no common superscript differ (*p* < 0.05).

The gut permeability was assessed by measuring the amounts of FITC-d in the serum of immunized birds. At 14 dpi the challenge group had a 27% increase in gut permeability, whereas the vaccine + challenge group reversed the increase in gut permeability by 13% (*p* < 0.05) (Figure 3).

### 3.7 The Effects of *Salmonella* CNP Vaccine on Gene Expression in the Cecal Tonsils or Jejunum of Vaccinated Birds

**FIGURE 4:**
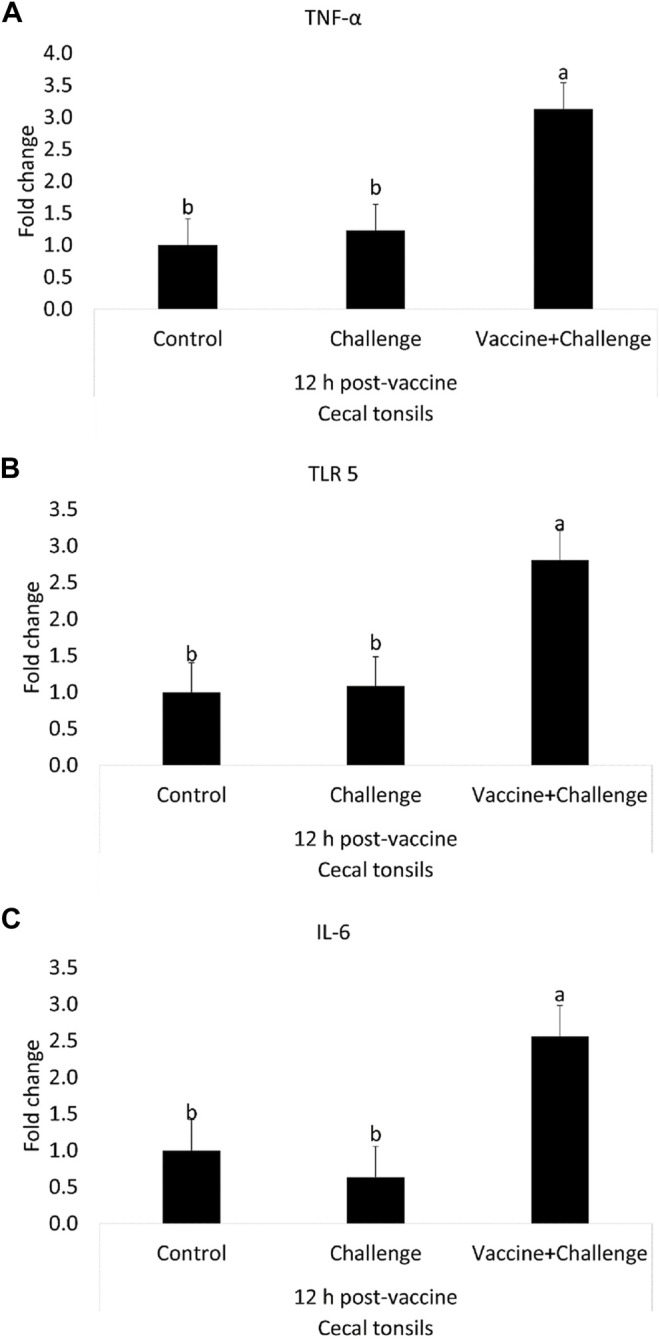
The Effects of *Salmonella* CNP Vaccine on Gene Expression in the Cecal Tonsils of Vaccinated Birds at 12 h post-vaccination. At d1 of age birds were allocated into treatment groups: 1) Control; 2) Challenge; or 3) Vaccine + Challenge. At d1 and d7 of age birds in the negative and positive control groups were mock vaccinated with PBS and birds in the treatment group were vaccinated with CNP. At d14 of age birds in the negative control group were given a mock challenge of 0.5 ml PBS/bird and birds in the positive control and the treatment group were orally challenged with 1 × 10^7^ CFU/bird of *S*. Enteritidis. Cecal tonsils were collected from one bird/pen (*n* = 6) at 12 h post-vaccination. Data represented as fold change compared to control. **(A)** TNF-α mRNA; **(B)** TLR 5 mRNA; **(C)** IL-6 mRNA. Bars (+SE) with no common superscript differ (*p* < 0.05).

At 12 h post-vaccination, the TNF-α mRNA amounts were 2-fold higher in the cecal tonsils of immunized birds when compared to control (*p* < 0.05) (Figure 4A). At 12 h post-vaccination, the TLR 5 mRNA amounts were 2-fold higher in the cecal tonsils of immunized birds when compared to control (*p* < 0.05) (Figure 4B). At 12 h post-vaccination, the IL-6 mRNA amounts were 2-fold higher in the cecal tonsils of immunized birds when compared to control (*p* < 0.05) (Figure 4C). At 12 h post-vaccination, there were no significant differences between any of the treatment groups for IFN-γ and IL-1β mRNA amounts in the cecal tonsils, compared to control (*p* > 0.05) (Supplementary Figure S5).

**FIGURE 5:**
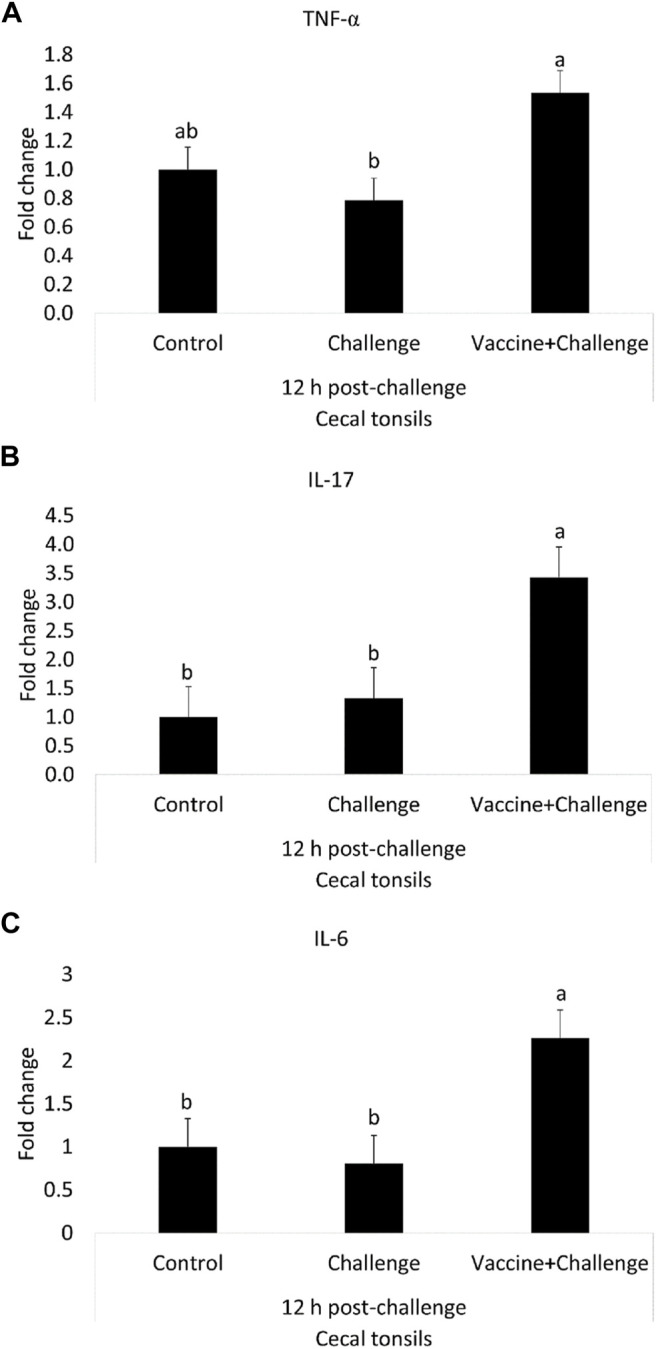
The Effects of *Salmonella* CNP Vaccine on Gene Expression in the Cecal Tonsils of Vaccinated Birds at 12 h post-challenge. At d1 of age birds were allocated into treatment groups: 1) Control; 2) Challenge; or 3) Vaccine + Challenge. At d1 and d7 of age birds in the negative and positive control groups were mock vaccinated with PBS and birds in the treatment group were vaccinated with CNP. At d14 of age birds in the negative control group were given a mock challenge of 0.5 ml PBS/bird and birds in the positive control and the treatment group were orally challenged with 1 × 10^7^ CFU/bird of *S*. Enteritidis. Cecal tonsils were collected from one bird/pen (n = 6) at 12 h post-challenge. Data represented as fold change compared to control. **(A)** TNF-α mRNA; **(B)** IL-17 mRNA; **(C)** IL-6 mRNA. Bars (+SE) with no common superscript differ (*p* < 0.05).

At 12 h post-challenge, the TNF-α mRNA amounts were 0.5-fold higher in the cecal tonsils of immunized birds when compared to control (*p* < 0.05) (Figure 5A). At 12 h post-challenge, the IL-17 mRNA amounts were 2-fold higher in the cecal tonsils of immunized birds when compared to control (*p* < 0.05) (Figure 5B). At 12 h post-challenge, the IL-6 mRNA amounts were 1.3-fold higher in the cecal tonsils of immunized birds when compared to control (*p* < 0.05) (Figure 5C). At 12 h post-challenge, there were no significant differences between any of the treatment groups for IFN-γ, TLR 4, IL-10, iNOS, K60, and TGF-β mRNA amounts in the cecal tonsils when compared to control (*p* > 0.05) (Supplementary Figure S6).

At 12 h post-challenge, there were no significant differences for Claudin-1 and Zona Occludens-1 mRNA amounts in jejunum samples, compared to control (*p* > 0.05) (Supplementary Figure S6).

At d 35 of age, there were no significant differences between any of the treatment groups for TGF-β, IL-10, IL-6, TNF-α, and IL-1β mRNA amounts in the cecal tonsils, compared to control (*p* > 0.05) (Supplementary Figure S7).

## 4 Discussion

Currently, polymeric chitosan nanoparticles are studied as antigen-delivery tools for killed *Salmonella* vaccine antigens. Previous studies with broiler and layer birds have investigated the efficacy of the oral *Salmonella* CNP vaccine when delivered by oral gavage, water, feed, *in-ovo*, and in a combined live followed by killed vaccination scheme (Acevedo-Villanueva et al., 2020; Renu, Han, et al., 2020; Renu, Markazi, et al., 2020; Y.; Han et al., 2020a; Yi; Han et al., 2020b; Acevedo-Villanueva et al., 2021; Acevedo-Villanueva, Renu and Gourapura, Renukaradhya, Selvaraj, 2021). Findings have demonstrated that the oral *Salmonella* CNP vaccine is a potential alternative to existing *Salmonella* vaccines for broilers and layers. When compared to uncoated CNPs and soluble OMP and flagellin antigens, previous studies demonstrated that flagellin-coated CNPs are readily uptaken by ileal Peyer Patch’s M cells by passing through mucus layer, elicited *Salmonella*-specific mucosal IgA and T cell responses and induced the expression of Th1 and Th2 cytokines mRNA expression (Renu, Han, et al., 2020). In this study, we assessed the efficacy of the *Salmonella* CNP vaccine delivered through gel-spray on broilers. All birds were vaccinated with PBS or CNP vaccine by gel-spray delivery at d1 of age and were given a booster vaccination of PBS or CNP vaccine at d7 of age by oral gavage. A shorter prime-boost interval was carried out because (1) unlike commercial breeders, broilers have a shorter life span, (2) *Salmonella* vaccines require two doses that are three to 4 weeks apart, and (3) *Salmonella* vaccines have a withdrawal period of 21 days before processing (Acevedo-villanueva et al., 2021). Thus, a shorter prime-boost approach takes into account the broiler’s short life span while complying with the 21-days withdrawal restriction. Previous research also explored the shorter prime-boost interval approach and found that the CNP can significantly reduce *S*. Enteritidis when it is orally delivered as a one-dose vaccination or as a booster vaccination (Acevedo-Villanueva et al., 2021).

In this study, we found that 87% of the antigens were successfully entrapped into the nanoparticle. To ensure the safe delivery of the antigens to the gut, we assessed the stability of the CNP vaccine at biological pH levels that range from the crop to the small intestine of broilers (Ravindran, 2013). According to literature, the vaccine must resist the pH from the crop to the gizzard at pH 3.5–5.5 to reach the small intestine intact at pH 6–7.5 (Gauthier, 2002). The delivery of the CNP vaccine antigens at the pH of the small intestine is essential to provide a stimulus to the immune system (Fredriksen and Grip, 2012). Results show that at pH 6.5 the CNP releases 31% of the antigen load, and at pH 7.5 the CNP releases 26% of the antigen load. The *in-vitro* results indicate that when the CNP vaccine is at the pH range of the small intestine, it would be releasing approximately 57% of its antigen load.

The average transit time of feed particles from the crop to the small intestine is approximately 70 min (Ravindran, 2013); hence, we assessed the cumulative protein release of antigens for the CNP at the initial stages after delivery for up to 17 h. The cumulative protein release of antigens for the CNP was determined to be 75% of its total protein after 17 h of incubation at a pH of 7.4. Previous research has reported that CNP loaded with Newcastle disease virus F-protein employed a slow release of 28% of their protein load at 18 h of incubation (Zhao et al., 2012). Another study reported that CNP loaded with native or toxoids of extracellular proteins of *Clostridium perfringens* and surface-tagged with *Salmonella* flagellin proteins had a cumulative release of approximately 10 and 16%, respectively, after 17 h of incubation (Akerele et al., 2020). Compared to other studies, our results show an increased cumulative protein release at 17 h of incubation. This difference could be due to the different TPP ratios among nanoparticle formulations, as it has been shown that as the TPP ratio increases in the vaccine formulation the protein loading efficiency decreases, and protein release from the nanoparticle increases (Hou et al., 2012).

When incubating the cRBCs with 20 μg/ml CNP and with 100 μg/ml CNP, the cRBCs released less than 1% and approximately 1% of hemoglobin, respectively, indicating no adverse effect of CNP on cRBCs. In previous research where CNP was loaded with native extracellular proteins of *Clostridium perfringens* and surface-tagged with *Salmonella* flagellin proteins, the CNP released less than 3% from cRBC (Akerele et al., 2020), further substantiating our results. The CNP loaded with Newcastle disease virus F-protein has also been demonstrated to be safe in chicken embryo kidney cells (Zhao et al., 2012).

*Salmonella* enterica serovars infections are not a problem to poultry health as they are asymptomatic carriers and look healthy during infection. The main concern with *Salmonella* and poultry infections arises when humans interact with infected live poultry or consume infected poultry meat or poultry-derived products and develop *Salmonellosis* (Centers for Disease Control and Prevention, 2021). However, in this study, all birds were monitored twice a day for dehydration, refusal to eat food, diarrhea, bloody feces, and lethargy during the experimental period. Further, production performance parameters were also monitored during the experimental period. As expected, the birds in the challenge and the vaccine + challenge groups showed no *Salmonella* symptoms during the experimental period. Moreover, the *S.* Enteritidis challenge had no significant effects on the BWG or FCR of the challenged birds during the experimental period. Our *in-vivo* study also found that CNP vaccination did not significantly affect BWG or FCR, as seen by the BWG and FCR of the birds that were vaccinated with the CNP vaccine and challenged with *S*. Enteritidis. Results are in accord with existing research where the CNP vaccine had no adverse effects on the production performance parameters of broilers or layers (Zhao et al., 2012; Acevedo-Villanueva et al., 2020; Akerele et al., 2020; Renu, Markazi, et al., 2020; Y.; Han et al., 2020a; Yi; Han et al., 2020b; Acevedo-Villanueva et al., 2021; Acevedo-Villanueva, Renu and Gourapura, Renukaradhya, Selvaraj, 2021).

Oral vaccination elicits a greater mucosal response through the production of substantial amounts of IgA against the pathogen of interest (Neutra and Kozlowski, 2006). In this study, the OMP IgY antibody titers in the serum of vaccinated birds significantly increased after the booster vaccination, when compared to the unvaccinated control birds. A significant antigen-specific IgY antibody increase can be seen as an immunological advantage, as indicated by various studies showing that IgG can also partake in the host defense against enteric pathogens (Holmgren and Czerkinsky, 2005; Brandtzaeg, 2009). Previous findings are in agreement with our results showing that the *Salmonella* CNP vaccine can elicit significant IgY antibody levels (Acevedo-Villanueva et al., 2020; Acevedo-Villanueva et al., 2021; Renu, Markazi, et al., 2020; Acevedo-Villanueva, Renu and Gourapura, Renukaradhya, Selvaraj, 2021).

IgA in the serum is actively and selectively transported from the blood to the bile by the liver (Davis and Sell, 1989). Further, mucosal IgA functions as the primary defense mechanism against enteric pathogens, like *Salmonella*. For this study, the OMP IgA and Flagellin IgA antibody titers in the bile of vaccinated birds significantly increased after the booster vaccination, when compared to the unvaccinated control birds. The CNP vaccine was able to induce significantly greater levels of antigen-specific antibodies against *S*. Enteritidis before the experimental challenge. Further, the OMP and Flagellin IgA antibody titers in the bile of vaccinated birds also had a significant increase in response to the *S.* Enteritidis challenge, when compared to the unvaccinated control birds. The significant increase in OMP IgA and Flagellin IgA antibody titers in the bile of vaccinated birds at d35 of age were from birds that had already significantly decreased the *S*. Enteritidis colonization. Their antigen-specific antibody titers were similar to that of the antibody titers observed in the unvaccinated-challenge birds. However, the unvaccinated-challenged birds were still struggling to clear the *S.* Enteritidis infection, as indicated by the significant increase of *S*. Enteritidis colonization. These findings indicate that the CNP vaccine can produce significant antigen-specific IgA titers, similar to that of infection, and can also aid in the clearance of *S*. Enteritidis in the gut. However, the primary mechanism of *Salmonella* clearance in the gut of the vaccinated birds seems to be directed towards the cell-mediated component of the immune response. This is relevant because killed vaccines elicit weak cell-mediated responses when compared to live vaccines (Wodi AP and Morelli V, 2021). However, our findings demonstrate that the CNP vaccine can elicit a significant cellular immune response, as seen by its capability to elicit a significant antigen-specific lymphocyte proliferation against *S.* Enteritidis flagellin antigens. For this reason, future research should further explore the cellular immune mechanisms behind the vaccine. Further, results are in agreement with previous research demonstrating that in chickens an infection with *Salmonella* enterica serovar Typhimurium induces high levels of antigen-specific antibodies, but B-cells do not play an essential role in the clearance of the primary infection (Beal et al., 2006). Currently, it is not clear what non-B cell mechanism mediates *Salmonella* clearance; which could be beneficial to developing a successful *Salmonella* vaccine for broilers. For this study, results demonstrated that the CNP vaccine can induce protective OMP and Flagellin IgA antibody titers in bile. Also, further research is needed to better understand the cellular and humoral advantages that the CNP can convey.

In chickens, cloacal swabs are an easy and reliable way to evaluate mucosal IgA concentration (Merino-Guzmán et al., 2017). For this study, the OMP and Flagellin IgA antibody titers in the cloacal swabs of vaccinated birds also had a significant increase in response to the *S*. Enteritidis challenge, when compared to the unvaccinated control birds. Results are in agreement with previous studies that have reported that the CNP vaccine can elicit significant antigen-specific IgA antibody titers against *S*. Enteritidis (Acevedo-Villanueva et al., 2020; Renu, Han, et al., 2020; Renu, Markazi, et al., 2020; Y.; Han et al., 2020a; Yi; Han et al., 2020b; Acevedo-Villanueva et al., 2021; Acevedo-Villanueva, Renu and Gourapura, Renukaradhya, Selvaraj, 2021).

An antigen-specific lymphocyte proliferation assay was carried out to evaluate the cell-mediated response of birds that are vaccinated with CNP against *S.* Enteritidis antigens. The CNP vaccine was surface-coated with a flagellin crude protein extract as an adjuvant that is capable of being recognized by the host TLR 5 and triggering the immune system. Our results indicate that 20 μg/ml of flagellin crude protein extract can induce a substantial antigen-specific proliferation response against *Salmonella*, making it is a suitable choice as an adjuvant for the CNP vaccine. This study also found that the TLR 5 mRNA expression was significantly upregulated at 12 h post-vaccination in birds that were immunized with the CNP vaccine. The observed results are in agreement with previous studies regarding the PBMCs recall response against loaded CNP antigen stimulation (Renu, Markazi, et al., 2020; Y.; Han et al., 2020b; Yi; Han et al., 2020c; Acevedo-Villanueva et al., 2021) and a previous study that assessed the upregulation of TLR 5 by the CNP vaccine (Renu, Han, et al., 2020). Further, the numerical differences (*p* > 0.05) in cell proliferation of birds in the control and the challenge groups at d12 of age can be due to individual variations. Variations among individuals of the same species can result in differences in the immune responses (Nature Education, 2014; Urban, 2020).

For this study, we also investigated if spleenocytes from birds that were vaccinated with the CNP, loaded with the *S*. Enteritidis antigens, can also recognize antigens from other prominent *Salmonella* enterica serovars. The *ex-vivo* assessment was done to study the CNP vaccine’s potential to provide cross-protection against heterologous *Salmonella* serovars. Cross-protection against heterologous *Salmonella* serovars requires vaccine antigens that share conserved sequences (Vojtek et al., 2019). Conserved sequences allow the immune system of the vaccinated birds to recognize multiple *Salmonella* serovars, which benefits the poultry industry. The *ex-vivo* results indicate that the T-lymphocytes from immunized birds responded in an antigen-specific manner when stimulated with 20 μg/ml of either *S*. Enteritidis, *S*. Typhimurium, or *S*. Litchfield HK. Results indicate that the CNP vaccine could significantly provide cross-protection against other *Salmonella* enterica serovars. The CNP vaccine was loaded with a crude-enriched extract of OMP and flagellin proteins from *S*. Enteritidis. It is expected that vaccines that are synthesized with either the Enteritidis or Typhimurium serovar can provide cross-protection against both because *S*. Enteritidis and *S*. Typhimurium porin OMP C contains several amino acid sequences that are highly conserved (Valero-Pacheco et al., 2020), making them suitable vaccine candidates. Further, *S*. Enteritidis and *S*. Litchfield have been linked to a massive multi-state outbreak in 2018 that was traced to live poultry (Robertson et al., 2019). Further research found that *S*. Enteritidis and *S*. Litchfield contain significant homology in their genetic sequence, making cross-protection possible during an *S.* Enteritidis vaccination (Robertson et al., 2019). Future research should consider the *in-vivo* study of the *Salmonella* CNP vaccine against experimental challenges of *S*. Typhimurium or *S*. Litchfield to fully explore the vaccine’s potential for cross-protection.

*Salmonella* control and prevention requires a coordinated and multifaceted approach with several intervention strategies of which vaccination is only one. Currently, *Salmonella* vaccines alone cannot fully eradicate *Salmonella* in poultry, but they are strongly recommended to significantly help reduce the *Salmonella* loads in poultry production. Early interventions at the live stage of the birds can significantly contribute to decreasing the *Salmonella* load at the market age of 35–49 days of age. A previous study identified that *S*. Enteritidis can colonize the spleen, liver, small intestine, cecum contents, stomach, heart, pancreas, and the blood of broilers during 21 days, from highest to lowest distribution (Zeng et al., 2018). For this reason, the current study aimed to explore the efficacy of the CNP vaccine in decreasing the colonization of *S*. Enteritidis in the internal organs of broilers at d35 of age. Broilers are asymptomatic carriers of non-typhoidal *Salmonella* (Shanmugasundaram et al., 2015), while healthy individuals require the presence of 1 × 10^6^ bacterial cells to cause infection (Alena Klochko, 2019). Thus, all birds in the challenge and the vaccine + challenge groups were given an oral challenge of 1 × 10^7^ CFU/bird of *S*. Enteritidis. Our findings demonstrate that the CNP vaccine can aid in significantly reducing the *S*. Enteritidis colonization by 2 Log in the ceca and spleen, and by 0.6 Log in the small intestine of immunized and experimentally challenged birds at d35 of age. A 2 Log reduction in two of the organs that have been reported to have the highest *S*. Enteritidis loads places the CNP as a viable vaccine candidate for *Salmonella* in poultry. Further, a significant 2 Log reduction of *Salmonella* at market age is of biological importance because combining the CNP vaccine with active “on-farm” and processing interventions can significantly reduce the number of contaminated carcasses. Future research should explore the vaccine’s potential to reduce the *Salmonella* load in ready-to-eat carcasses of broilers.

Gut integrity is a crucial indicator of both health and critical illness, as damage to the intestinal epithelia induces “hyper-permeability” that can lead to bacterial translocation and subsequent systemic infection (Otani and Coopersmith, 2019). In this study, results indicate that the CNP vaccine treatment significantly reduced the loss in gut permeability, as seen by decreased levels of FITC-d in the serum of birds that were vaccinated with CNP. Results were further confirmed by the significant increase in FITC-d levels in the serum of birds in the challenge group when compared to control. Additionally, the mRNA expression levels of both Claudin-1 and Zona Occludens-1 at 12 h post-challenge were decreased (*p* > 0.05) in the jejunum samples of the challenge group. Previous findings demonstrate that *S.* Typhimurium is known to increase intestinal permeability of the chicken gut due to its ability to induce tight junction protein damage, as seen by the decreased expression of a few key markers, such as Claudin-1 and Zona Occludens-1 (Tafazoli, Magnusson and Zheng, 2003; Wang et al., 2018; Leyva-Diaz et al., 2021). Further, a study with rats found that *S*. Enteritidis can increase gut permeability as the result of environmental stress which can increase the *Salmonella* virulence factors (Ten Bruggencate et al., 2005). Future research should further explore the impact of CNP in the gut permeability of chickens that are challenged with *S.* Enteritidis.

For this study, we explored the CNP vaccine effect on key immune-related gene expressions of biological relevance to *Salmonella* infections in broilers. The induction of immune relevant cytokines and proteins, such as IL-1β, TNF-α, IFN-γ, IL-6, IL-10, IL-17, TLR 4, TLR 5, iNOS, TGF-β, K 60, Claudin-1, and Zona Occludens-1, following *Salmonella* infection of chickens have been studied previously (Van Immerseel et al., 2002; Withanage et al., 2004; Berndt et al., 2007; Ivanov et al., 2008; Crhanova et al., 2011; He, Genovese and Kogut, 2011; Matulova et al., 2012; Renu, Markazi, et al., 2020; Shanmugasundaram et al., 2021). The ability of a vaccine to induce significant levels of pro-inflammatory cytokines upon vaccination mimics the natural inflammatory response seen in the initial stages of infection, which is key to successfully triggering an immune response against the desired antigen. In mammals, the synthesis and release of pro-inflammatory cytokines, such as TNF-α and IL-6, are increased especially during the early stages of inflammation (Abbas Abul, 2014). Further, TNF-α and IL-6 are pleiotropic cytokines, so they can act as both pro-inflammatory and anti-inflammatory. In this study, birds that were immunized with the CNP vaccine had significantly higher mRNA expression of TNF-α and IL-6 at 12 h post-vaccination and 12 h post-challenge. These results highlight the ability of the CNP vaccine to induce a significant pro-inflammatory response against the loaded antigens, similar to that which is observed in a *Salmonella* infection. Results are in agreement with *in-vitro* findings that report that CNP vaccines can elicit increased secretion of TNF-α and IL-6 in immunized pig cells (Dhakal et al., 2018). Future studies will also explore the mRNA levels of key cytokines in serum samples of immunized birds.

IL-17 is predominantly produced by T helper 17 (Th17) cells (Xu and Cao, 2010). There is a significant gap in research regarding Th17 cells and *Salmonella* in chickens, and the research to date is contradicting. Further, there is no research regarding CNP effects in IL-17 cells or Th17 cytokines in chickens challenged with *Salmonella*. According to literature, Th17 cells and Th17 cytokines play an important role in the resistance to mucosal *Salmonella* infections across different species (Raffatellu M, George MD, Akiyama Y, 2009; Griffin and McSorley, 2011), including chickens (Crhanova et al., 2011). It has also been reported that chicken IL-17RA expression remains unchanged in *Salmonella* infection (Kim et al., 2014). However, in this study, our findings show that the birds that were immunized with the CNP vaccine had significantly higher IL-17 mRNA expression in response to the *S*. Enteritidis challenge when compared to the control groups. At 12 h post-challenge, both the control and the challenge groups had similar and significantly lower levels of IL-17. Thus, we hypothesize that the increased levels of Th17 mRNA can be due to a synergistic effect of the vaccine-loaded antigens and the increased IL-6 mRNA amounts induced by the CNP vaccine. Our hypothesis is based on previous research in mice that indicates that *Salmonella*-specific Th17 cells can recognize *Salmonella* flagellin (Lee et al., 2012) and that *Salmonella* Flagellin antigen has also shown an intrinsic ability to elicit IL-1 and IL-6 production (Salazar-Gonzalez and McSorley, 2005; Mizel and Bates, 2010). Moreover, it has been reported that IL-6 is a non-redundant differentiation factor for Th17 cells (Korn and Hiltensperger, 2021) in which significantly increased levels of IL-6 can promote the generation of Th17 cells (Hou et al., 2014) and result in IL-6 and IL-17 cytokines. So, the recognition of the vaccine’s flagellin by TLR 5 and its ability to induce significant IL-6 mRNA may be the force driving the Th17 response against *Salmonella* in the cecal tonsils during *S*. Enteritidis infection in chickens. These results also support the hypothesis that the primary mechanism of *Salmonella* clearance in the gut is due to the cellular component of the immune response (Beal et al., 2006). Future research regarding the effect of the CNP vaccine on *Salmonella* Th17 cells and IL-17 cytokines is needed to explore the IL-17 and *Salmonella* research gap in chickens.

## 5 Conclusion

This study analyzed the efficacy of a CNP vaccine against *Salmonella* delivered by gel-spray to broilers. The CNP vaccine (1) can overcome the hurdles of conventional vaccines; (2) had no negative effects on production performance of broilers; (3) elicited antigen-specific systemic and mucosal immune responses; (4) elicited antigen-specific recall response against *S*. Enteritidis Flagellin, *S*. Enteritidis HKA, *S*. Typhimurium HKA, and S. Litchfield HKA; (5) reduced the *Salmonella* load in the ceca, spleen and small intestine of broilers at d35 of age; (6) decreased the loss in gut permeability caused by *S*. Enteritidis; and (7) increased IL-6, IL- 17, and TNF-α mRNA in cecal tonsils. Future studies will examine the CNP vaccine potential to convey cross-protection against heterologous *Salmonella* serovars, the efficacy of the CNP vaccine in reducing *S*. Enteritidis loads in ready-to-eat carcasses, and the relationship between the CNP vaccine and the Th17/IL-17 immune response to explore the role of Th17 cells and IL-17 cytokines on *Salmonella* infections in broilers. We conclude that the CNP vaccine is a viable alternative to conventional *Salmonella* vaccines for poultry.

## Data Availability

The original contributions presented in the study are included in the article/Supplementary Material, further inquiries can be directed to the corresponding author.
