# Supplementary material for: Immunization of Broiler Chickens With a Killed Chitosan Nanoparticle Salmonella Vaccine Decreases Salmonella Enterica Serovar Enteritidis Load
Source: Front Physiol. 2022 Jul 18;13:920777. doi: 10.3389/fphys.2022.920777 (PMC9340066; doi:10.3389/fphys.2022.920777)
Supplement: Supplementary file 2 [file Image6.pdf]

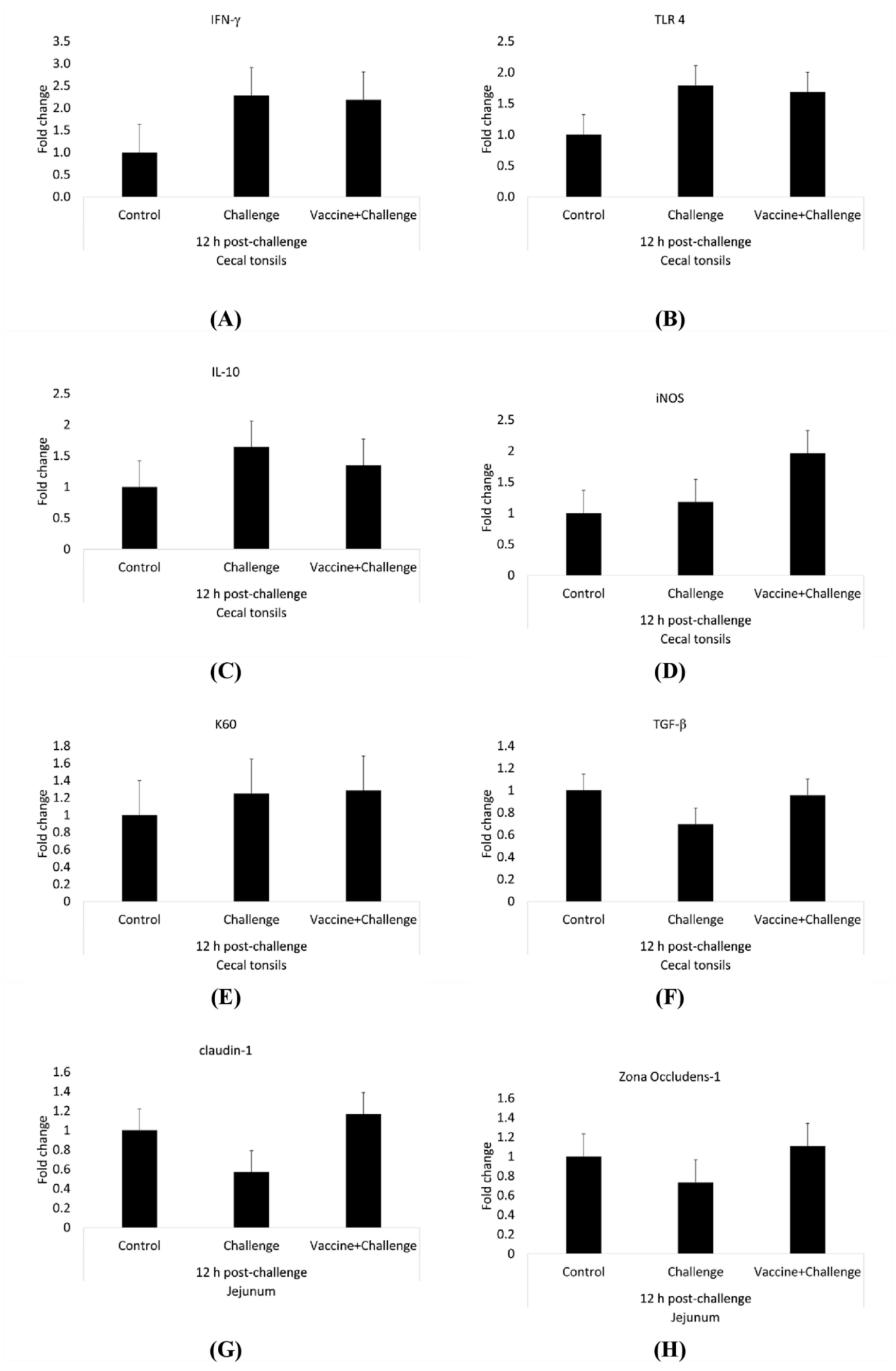
IFN-γ
Fold change
3.5
3.0
2.5
2.0
1.5
1.0
0.5
0.0
Control
Challenge
Vaccine+Challenge
12 h post-challenge
Cecal tonsils
(A)
TLR 4
Fold change
2.5
2.0
1.5
1.0
0.5
0.0
Control
Challenge
Vaccine+Challenge
12 h post-challenge
Cecal tonsils
(B)
IL-10
Fold change
2.5
2
1.5
1
0.5
0
Control
Challenge
Vaccine+Challenge
12 h post-challenge
Cecal tonsils
(C)
iNOS
Fold change
2.5
2
1.5
1
0.5
0
Control
Challenge
Vaccine+Challenge
12 h post-challenge
Cecal tonsils
(D)
K60
Fold change
1.8
1.6
1.4
1.2
1
0.8
0.6
0.4
0.2
0
Control
Challenge
Vaccine+Challenge
12 h post-challenge
Cecal tonsils
(E)
TGF-β
Fold change
1.4
1.2
1
0.8
0.6
0.4
0.2
0
Control
Challenge
Vaccine+Challenge
12 h post-challenge
Cecal tonsils
(F)
claudin-1
Fold change
1.6
1.4
1.2
1
0.8
0.6
0.4
0.2
0
Control
Challenge
Vaccine+Challenge
12 h post-challenge
Jejunum
(G)
Zona Occludens-1
Fold change
1.6
1.4
1.2
1
0.8
0.6
0.4
0.2
0
Control
Challenge
Vaccine+Challenge
12 h post-challenge
Jejunum
(H)

Supplementary Figure 6. The Effects of *Salmonella* CNP Vaccine on Gene Expression in the Cecal Tonsils or Jejunum of Vaccinated Birds at 12 h post-challenge. At d1 of age birds were allocated into treatment groups: 1) Control; 2) Challenge; or 3) Vaccine + Challenge. At d1 and d7 of age birds in the negative and positive control groups were mock vaccinated with PBS and birds in the treatment group were vaccinated with CNP. At d14 of age birds in the negative control group were given a mock challenge of 0.5 mL PBS/bird and birds in the positive control and the treatment group were orally challenged with 1×10^7 CFU/bird of *S*. Enteritidis. Cecal tonsils and jejunum samples were collected from one bird/pen (n=6) at 12 h post-challenge. Data represented as fold change compared to control. **(A)** IFN-γ mRNA; **(B)** TLR 4 mRNA; **(C)** IL-10 mRNA; **(D)** iNOS mRNA; **(E)** K60 mRNA; **(F)** TGF-β mRNA; **(G)** Claudin-1 mRNA; **(H)** Zona Occludens-1 mRNA. Bars (+SE) with no common superscript differ ($P<0.05$).
