# Supplementary material for: Immunization of Broiler Chickens With a Killed Chitosan Nanoparticle Salmonella Vaccine Decreases Salmonella Enterica Serovar Enteritidis Load
Source: Front Physiol. 2022 Jul 18;13:920777. doi: 10.3389/fphys.2022.920777 (PMC9340066; doi:10.3389/fphys.2022.920777)
Supplement: Supplementary file 3 [file Image4.pdf]

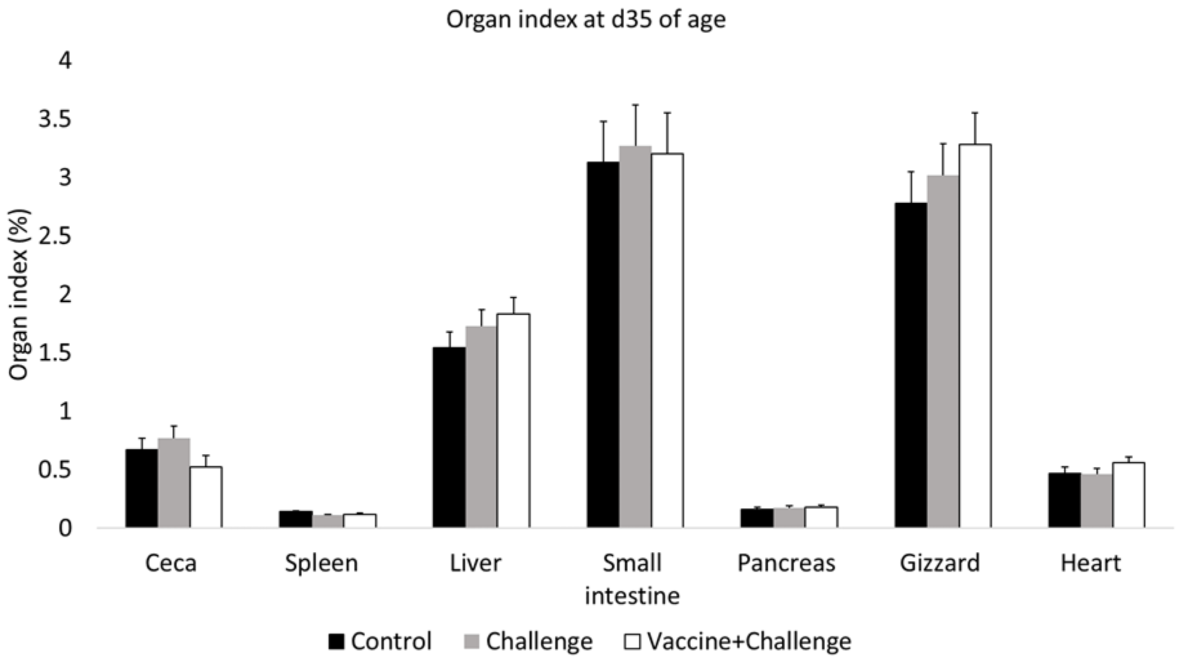

Supplementary Figure 4. The effect of CNP vaccine on the relative weight of different organs. At d1 of age birds were allocated into treatment groups: 1) Control; 2) Challenge; or 3) Vaccine + Challenge. At d1 and d7 of age birds in the negative and positive control groups were mock vaccinated with PBS and birds in the treatment group were vaccinated with CNP. At d14 of age birds in the negative control group were given a mock challenge of 0.5 mL PBS/bird and birds in the positive control and the treatment group were orally challenged with 1×10^7 CFU/bird of *S*. Enteritidis. Gizzard, pancreas, small intestine, spleen, liver, ceca, heart, and blood samples were collected from one bird/pen (n=6) at 21 dpi and analyzed for *S*. Enteritidis loads by plating. The organ weight was further used to observe the CNP vaccine effect on the relative weight of different organs by calculating the organ index as Weight Index (%) = (organ weight (g)) / (live weight (g)) × (100).
