# Supplementary material for: Immunization of Broiler Chickens With a Killed Chitosan Nanoparticle Salmonella Vaccine Decreases Salmonella Enterica Serovar Enteritidis Load
Source: Front Physiol. 2022 Jul 18;13:920777. doi: 10.3389/fphys.2022.920777 (PMC9340066; doi:10.3389/fphys.2022.920777)
Supplement: Supplementary file 4 [file Image2.pdf]

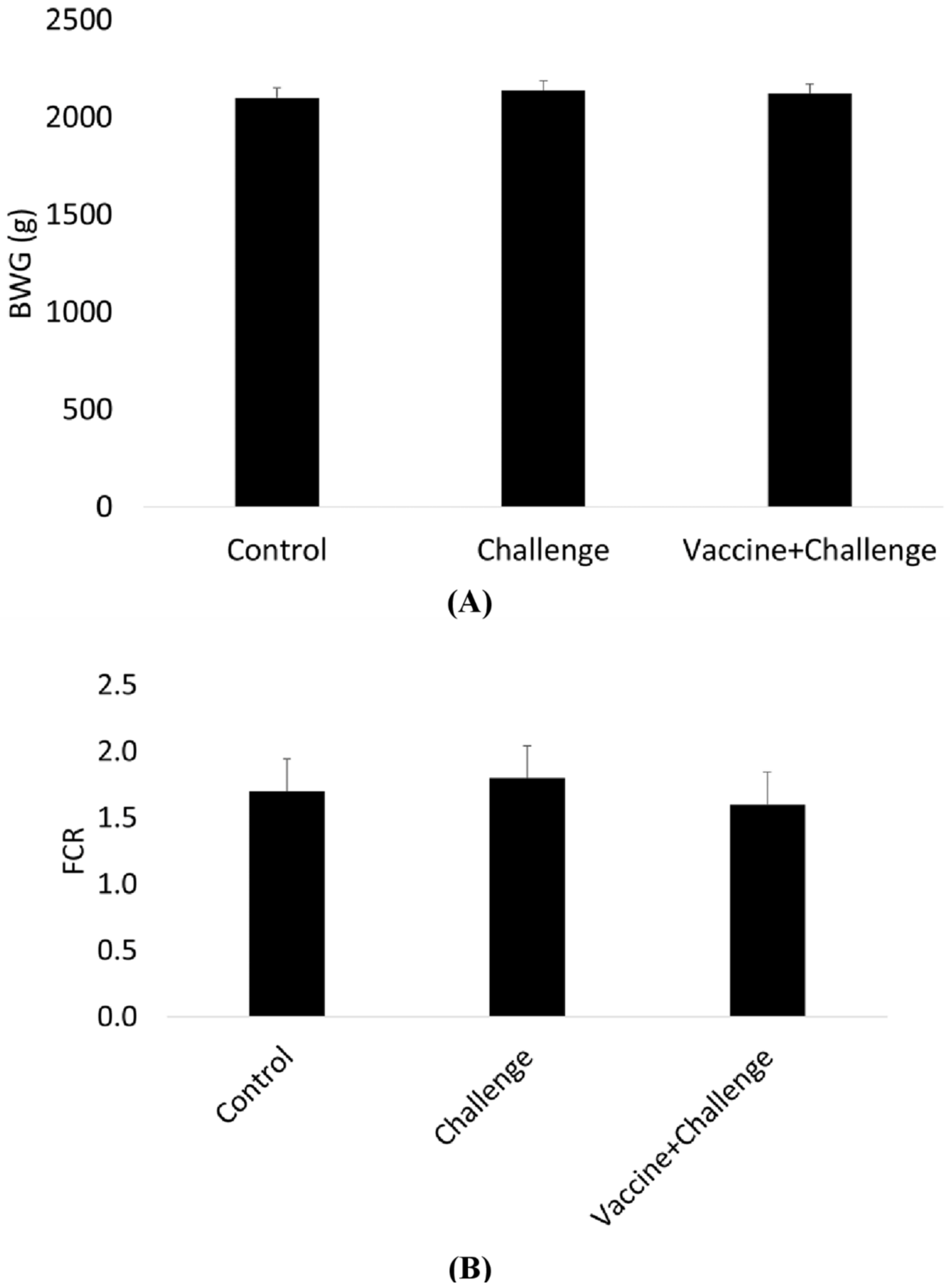

Supplementary Figure 2. The effects of *Salmonella* CNP vaccine on production performance of vaccinated birds. Data represents 6 pens per treatment. Results were reported as cumulative **(A)** BWG and **(B)** FCR from d0 to d35 of age. Bars (+SE) with no common superscript differ (P<0.05).
