# Supplementary material for: Immunization of Broiler Chickens With a Killed Chitosan Nanoparticle Salmonella Vaccine Decreases Salmonella Enterica Serovar Enteritidis Load
Source: Front Physiol. 2022 Jul 18;13:920777. doi: 10.3389/fphys.2022.920777 (PMC9340066; doi:10.3389/fphys.2022.920777)
Supplement: Supplementary file 5 [file Image3.pdf]

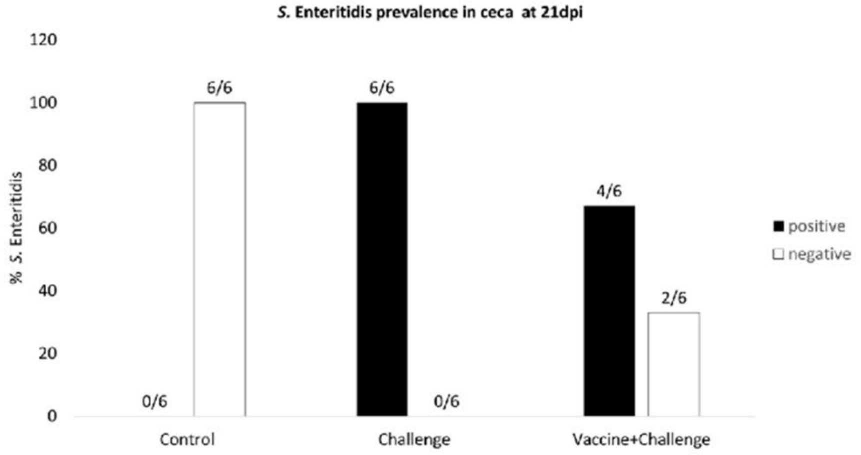

S. Enteritidis prevalence in ceca at 21dpi
% S. Enteritidis
120
100
80
60
40
20
0
0/6
6/6
6/6
0/6
4/6
2/6
Control
Challenge
Vaccine+Challenge
positive
negative

(A)

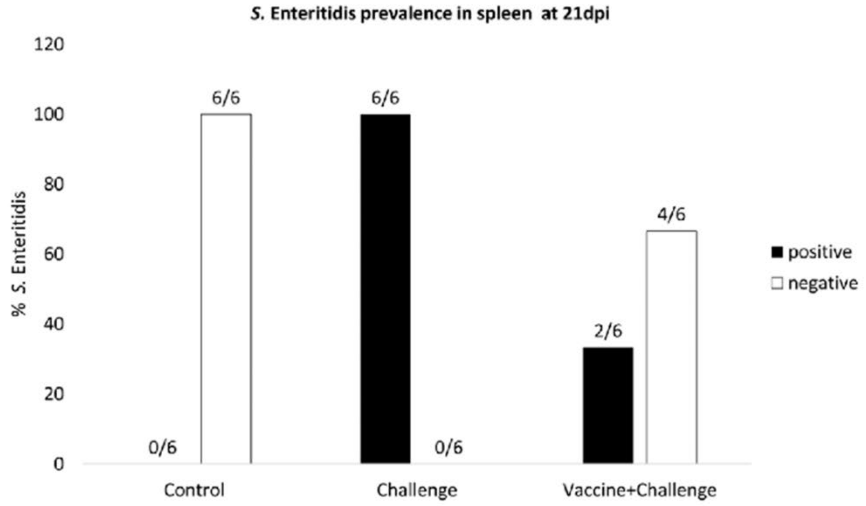

S. Enteritidis prevalence in spleen at 21dpi
% S. Enteritidis
120
100
80
60
40
20
0
0/6
6/6
6/6
0/6
2/6
4/6
Control
Challenge
Vaccine+Challenge
positive
negative

(B)

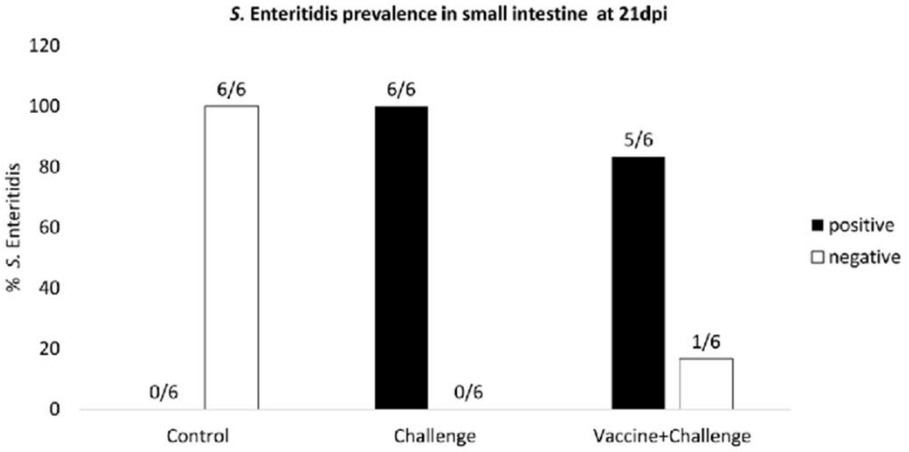

S. Enteritidis prevalence in small intestine at 21dpi
% S. Enteritidis
120
100
80
60
40
20
0
0/6
6/6
6/6
0/6
5/6
1/6
Control
Challenge
Vaccine+Challenge
positive
negative

(C)

Supplementary Figure 3. Prevalence of *S.* Enteritidis in ceca, spleen, and small intestine of vaccinated birds. At d1 of age birds were allocated into treatment groups: 1) No vaccine, no challenge; 2) No vaccine, challenge; or 3) Vaccine, booster, challenge. At d1 and d7 of age birds in the negative and positive control groups were mock vaccinated with PBS and birds in the treatment group were vaccinated with CNP. At d14 of age birds in the negative control group were given a mock challenge of 0.5 mL PBS/bird and birds in the positive control and the treatment group were orally challenged with 1×10^7 CFU/bird of *S.* Enteritidis. Gizzard, pancreas, small intestine, spleen, liver, ceca, heart, and blood samples were collected from one bird/pen (n=6) at 21 dpi and analyzed for *S.* Enteritidis loads by plating. *Salmonella* Enteritidis was detected in the **(A)** ceca, **(B)** spleen, and **(C)** small intestine of vaccinated birds. Samples that were double negative for *S.* Enteritidis presence after selective enrichment were considered negative for *Salmonella* colonization.
