# Supplementary material for: Immunization of Broiler Chickens With a Killed Chitosan Nanoparticle Salmonella Vaccine Decreases Salmonella Enterica Serovar Enteritidis Load
Source: Front Physiol. 2022 Jul 18;13:920777. doi: 10.3389/fphys.2022.920777 (PMC9340066; doi:10.3389/fphys.2022.920777)
Supplement: Supplementary file 6 [file Image7.pdf]

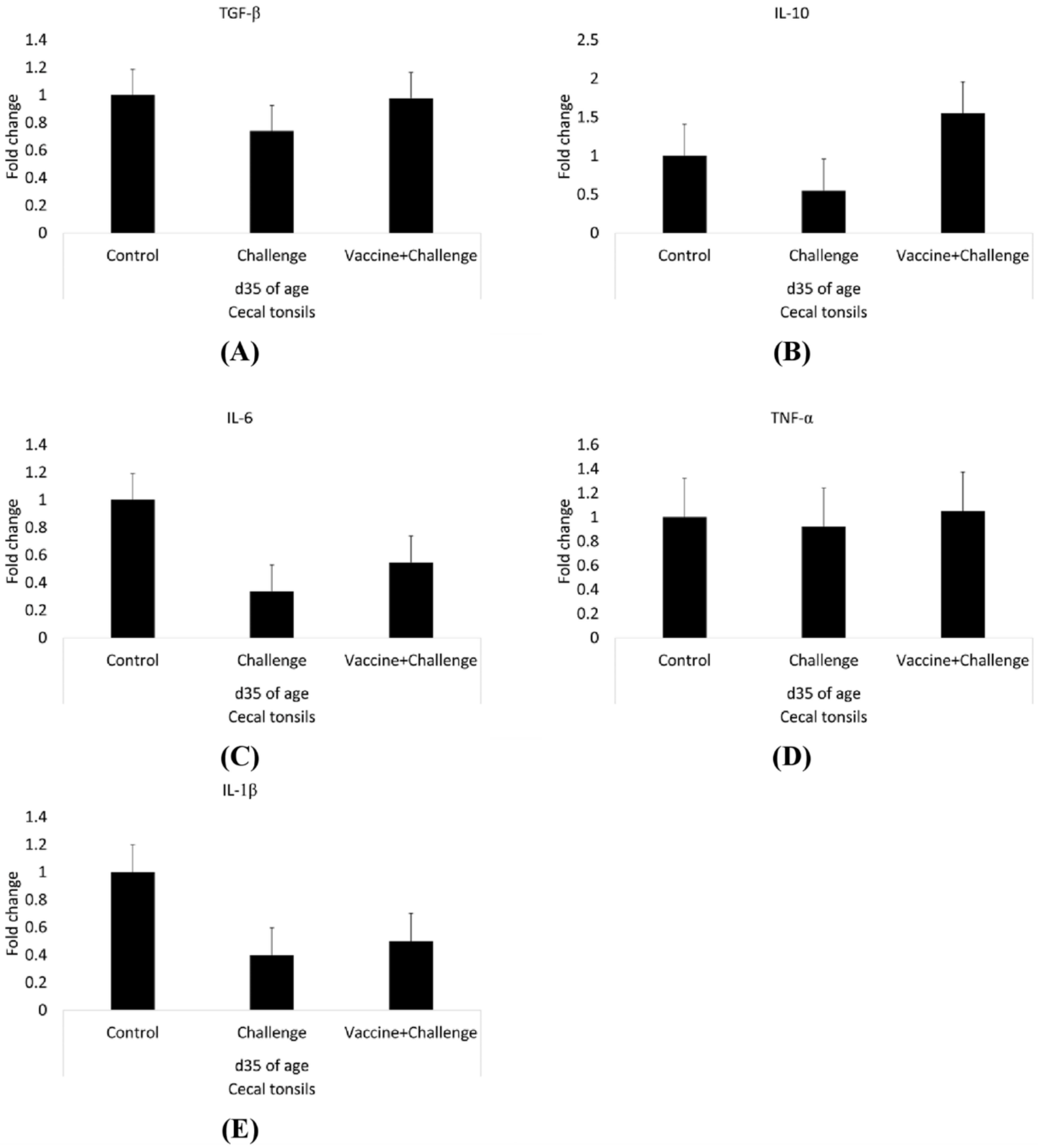

Supplementary Figure 7. The Effects of *Salmonella* CNP Vaccine on Gene Expression in the Cecal Tonsils of Vaccinated Birds at d35 of age. At d1 of age birds were allocated into treatment groups: 1) Control; 2) Challenge; or 3) Vaccine + Challenge. At d1 and d7 of age birds in the negative and positive control groups were mock vaccinated with PBS and birds in the treatment group were vaccinated with CNP. At d14 of age birds in the negative control group were given a mock challenge of 0.5 mL PBS/bird and birds in the positive control and the treatment group were orally challenged with 1×10^7 CFU/bird of *S.* Enteritidis. Cecal tonsils were collected from one bird/pen (n=6) at 12 d35 of age. Data represented as fold change compared to control. **(A)** TGF-β mRNA; **(B)** IL-10 mRNA; **(C)** IL-6 mRNA; **(D)** TNF-α mRNA; **(E)** IL-1β mRNA. Bars (+SE) with no common superscript differ (P<0.05).
