# Supplementary material for: Immunization of Broiler Chickens With a Killed Chitosan Nanoparticle Salmonella Vaccine Decreases Salmonella Enterica Serovar Enteritidis Load
Source: Front Physiol. 2022 Jul 18;13:920777. doi: 10.3389/fphys.2022.920777 (PMC9340066; doi:10.3389/fphys.2022.920777)
Supplement: Supplementary file 7 [file Image1.pdf]

frontiers

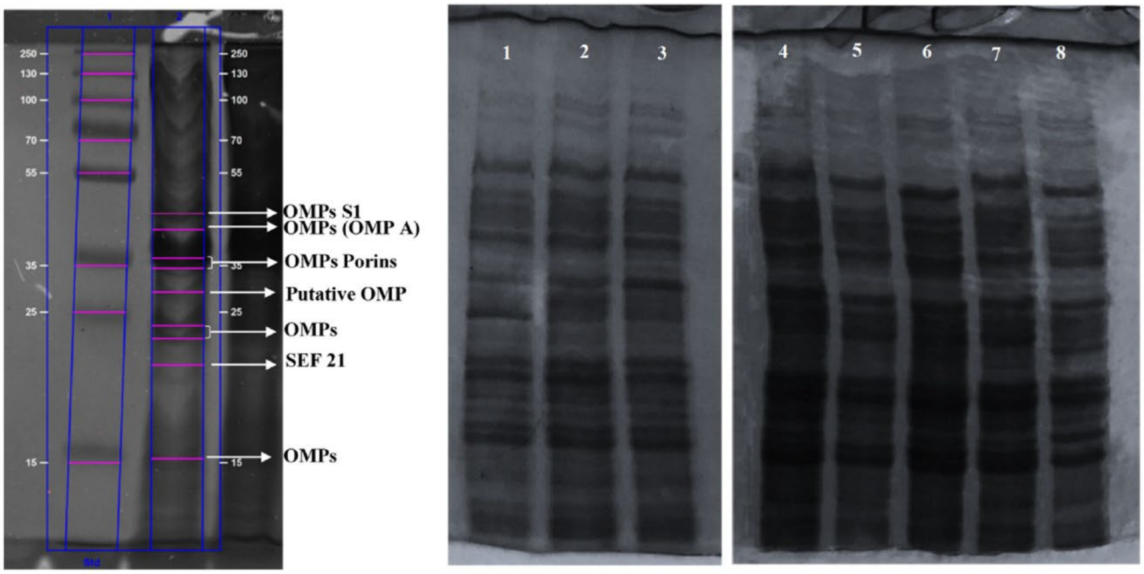

(A) (B)

Supplementary Figure 1. (A) SDS-PAGE analyses of *S*. Enteritidis-OMPs crude extract. Lane 1. Standard protein marker (kDa; Precision Plus Protein™ Standards, BioRad Inc.). Lane 2. S. Enteritidis OMPs. Proteins with approximate molecular weight 15, 21, 23, 24, 28, 35, 37, and 43 kDa were detected in the OMPs-enriched extract. Among them, the major well-characterized antigenic proteins were identified: These proteins were 43 kDa OmpS1, 37 kDa OmpA, 36 kDa OpmC, 35 kDa OmpF, 28 kDa putative Omp, 23 kDa Omp W, 21 kDa SEF21, and 15kDa OMPs (1,2). Image LabTM software was used for visualizing bands. **(B) SDS-PAGE analyses of *S*. Enterica serovars heat-killed whole-antigenic crude extract.** Outer membrane protein bands were visualized in the heat-killed whole antigenic crude extract of *Salmonella* Enterica serovars: Lane 1. *S*. Hadar, Lane 2. *S*. Litchfield, Lane 3. *S*. Newport, Lane 4. *S*. Enteritidis, Lane 5. *S*. Heidelberg, Lane 6. *S*. Typhimurium, Lane 7, *S*. Infantis, Lane 8. *S*. Kentucky.
